# Opposing activities of the Ras and Hippo pathways converge on regulation of YAP protein turnover

**DOI:** 10.15252/embj.201489385

**Published:** 2014-09-01

**Authors:** Xin Hong, Hung Thanh Nguyen, Qingfeng Chen, Rui Zhang, Zandra Hagman, P Mathijs Voorhoeve, Stephen M Cohen

**Affiliations:** 1 Institute of Molecular and Cell BiologySingapore City, Singapore; 2 Department of Biological Sciences, National University of SingaporeSingapore City, Singapore; 3 Duke-NUS Graduate Medical SchoolSingapore City, Singapore; 4 Singapore-MIT Alliance for Research, and TechnologySingapore City, Singapore; 5 Department of Genetics, Stanford UniversityPalo Alto, CA, USA; 6 Department of Cellular and Molecular Medicine, University of CopenhagenCopenhagen, Denmark

**Keywords:** anchorage independence, Hippo pathway, primary cell transformation, RasV12, tumor suppressor

## Abstract

Cancer genomes accumulate numerous genetic and epigenetic modifications. Yet, human cellular transformation can be accomplished by a few genetically defined elements. These elements activate key pathways required to support replicative immortality and anchorage independent growth, a predictor of tumorigenesis *in vivo*. Here, we provide evidence that the Hippo tumor suppressor pathway is a key barrier to Ras-mediated cellular transformation. The Hippo pathway targets YAP1 for degradation via the βTrCP-SCF ubiquitin ligase complex. In contrast, the Ras pathway acts oppositely, to promote YAP1 stability through downregulation of the ubiquitin ligase complex substrate recognition factors SOCS5/6. Depletion of SOCS5/6 or upregulation of YAP1 can bypass the requirement for oncogenic Ras in anchorage independent growth *in vitro* and tumor formation *in vivo*. Through the YAP1 target, Amphiregulin, Ras activates the endogenous EGFR pathway, which is required for transformation. Thus, the oncogenic activity of Ras^V12^ depends on its ability to counteract Hippo pathway activity, creating a positive feedback loop, which depends on stabilization of YAP1.

See also: **V Corbo et al** (November 2014)

## Introduction

The importance of the Hippo tumor suppressor pathway in human cancer has been recognized increasingly in recent years (reviewed in Harvey *et al*, 2013; Pan, 2010). Regulation of the activity of the transcriptional co-activators YAP and TAZ is the key output of the Hippo pathway. YAP and TAZ bind to transcription factors including TEAD and β-catenin to regulate genes required for cell proliferation and survival (Zhao *et al*, 2008; Heallen *et al*, 2011). Mutations in upstream Hippo pathway components that lead to YAP/TAZ activation have been identified in human cancers (Harvey *et al*, 2013). YAP/TAZ are overexpressed in numerous cancers (http://www.cBioportal.org; e.g., Zhang *et al*, 2009). Overexpression of YAP is sufficient to lead to tumor formation (Dong *et al*, 2007; Cai *et al*, 2010). High TAZ expression indicates a poor prognosis in colorectal cancer (Yuen *et al*, 2013). Therapeutic approaches to target the Hippo pathway are of growing interest (Liu *et al*, 2012; Liu-Chittenden *et al*, 2012; Johnson & Halder, 2014). In light of the importance of YAP/TAZ activity in human cancers, it will be important to develop a more complete understanding of the mechanisms that regulate YAP/TAZ activity.

The core of the Hippo pathway is a kinase cassette containing MST1/2 (Hippo in *Drosophila*) and LATS1/2. Hippo/MST kinase activity acts via a membrane-associated complex containing the tumor suppressor NF2/Merlin (Yin *et al*, 2013). MST kinases activate LATS kinases, which in turn phosphorylate the transcriptional co-activators YAP and TAZ on multiple sites, leading to their inactivation through cytoplasmic sequestration and increased degradation (Dong *et al*, 2007; Zhao *et al*, 2007, 2010; Hao *et al*, 2008; Lei *et al*, 2008). Phosphorylation of YAP and TAZ by LATS1/2 creates a priming site for casein kinase 1-mediated phosphorylation of nearby residues. CK1 phosphorylation creates the binding site for βTrCP, targeting YAP and TAZ for degradation by the βTrCP-SCF ubiquitin ligase complex (Liu *et al*, 2010; Zhao *et al*, 2010).

Here, we report a mechanism by which Ras controls YAP protein turnover independently of the Hippo pathway. Ras acts through downregulation of SOCS-box proteins, which serve as substrate recognition modules of Elongin B/C ubiquitin ligase complexes (Linossi & Nicholson, 2012). The transforming activity of oncogenic Ras^V12^ depends on its ability to downregulate SOCS-box proteins and thereby stabilize YAP. We identify SOCS6 as a substrate recognition factor targeting YAP for degradation. SOCS6 levels are low in several cancers, where they show a negative correlation with the levels of the YAP target Amphiregulin. Upregulation of Amphiregulin completes a feedback loop through which oncogenic Ras activates endogenous EGF Receptors during cellular transformation. Thus, the transforming potential of the Ras pathway appears to be mediated in part through bypassing the activity of the Hippo tumor suppressor pathway at the level of YAP protein turnover.

## Results

### Downregulation of SOCS5/6 is required for Ras-mediated cellular transformation

**Figure 1:**
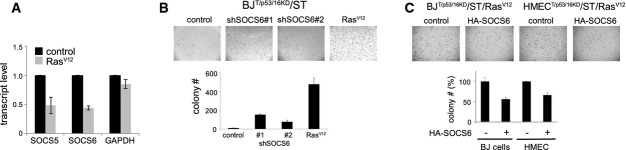
Downregulation of SOCS6 is required for Ras-mediated cellular transformation Effect of Ras^V12^ expression on *SOCS5* and *SOCS6* mRNA levels. BJ^T/p53/p16KD^/ST cells were transduced to express Ras^V12^ or with empty vector as a control. *P* < 0.05 comparing RNA levels in control and Ras^V12^-expressing cells (two-tailed Student's *t*-test). *GAPDH* mRNA was used as a control.Effect of SOCS5/6 depletion on soft agar colony formation. BJ^T/p53p16KD^/ST cells were transduced to express shRNAs targeting SOCS5/6 or Ras^V12^. Two independent shRNAs were used. Colony number: average of three independent experiment ± SD. *t*-test (two-tailed unequal variance): *P* < 0.05 comparing sh#1 or sh#2 to the vector control.Effect of SOCS6 overexpression on soft agar colony formation. BJ^T/p53/p16KD^/ST/ER-Ras^V12^ cells and HMEC^T/p53/p16KD^/ST/ER-Ras^V12^ cells were transduced to express SOCS6 or with empty vector as a control. Colony number: average of three independent experiments shown as % of the respective control ± SD. *P* < 0.05 comparing HA-SOCS6-expressing cells to their respective controls. SOCS6 and YAP immunoblot data for this experiment are shown in Fig3D.

Previously, we reported that the *Drosophila* SOCS-box protein, Socs36E, potentiates EGFR-driven tumor formation and metastasis (Herranz *et al*, 2012). Depletion of human SOCS5, the nearest ortholog of SOCS36E, increased the efficiency of Ras-mediated transformation of primary human cells, while having little or no effect on its own (Herranz *et al*, 2012). In the course of further study, we observed that expression of H-Ras^G12V^ (henceforth, Ras^V12^) reduced the level of *SOCS5* and *SOCS6* mRNAs (1A; similar result were obtained with K-Ras^G12V^ and N-Ras^G12V^, Supplementary Fig S1). To assess the contribution of SOCS6 to Ras-mediated transformation, we prepared vectors expressing shRNAs to target SOCS6. In controls for the efficacy of these shRNAs, we noted that depletion of SOCS6 also lowered *SOCS5* mRNA levels in BJ and HEK293T cells, whereas the SOCS5 shRNAs had little or no effect on SOCS6 levels (Supplementary Fig S2). In contrast to what we observed previously for SOCS5, co-depletion of SOCS5/6 proved to be sufficient to support anchorage independent growth and colony formation in soft agar (Fig1B). Polyclonal pools of primary human BJ fibroblasts were partially transformed by expression of hTert, p53- and p16- shRNAs, the SV40 small T oncogene. As shown previously (Hahn *et al*, 2002; Voorhoeve *et al*, 2006), these cells were capable of anchorage independent growth when expressing Ras^V12^ (Fig1B; similar result were obtained with K-Ras^G12V^ and N-Ras^G12V^, Supplementary Fig S1). shRNA-mediated depletion of SOCS5/6 was sufficient to induce colony formation in the absence of Ras^V12^ (Fig1B). Reciprocally, SOCS6 overexpression reduced soft agar colony formation by fully transformation-competent BJ^T/p53KD/p16KD/ST^ cells expressing Ras^V12^ (Fig1C). Comparable results were obtained using primary human mammary epithelial cells (HMEC, Fig1C). SOCS5/6 depletion also proved to be sufficient to support xenograft tumor formation by BJ^T/p53KD/p16KD/ST^ cells *in vivo*, without Ras^V12^ (Table1, Supplementary Fig S3). These experiments provide evidence that reducing SOCS5/6 activity is sufficient to replace oncogenic Ras and that downregulation of SOCS5/6 is required for Ras-mediated transformation of primary human cells.

**Table 1:** Tumor formation in immunocompromised mice

BJ^T/p53/p16KD/^ST +	No. of tumors/No. of injected	
	6.5 weeks	9 weeks
YAP^S127A^	6/6	Culled
Ras^V12^	6/6	Culled
shLats2 no. 1	0/6	3/6
shSOCS6 no. 2	0/6	4/6
Control	0/4	0/4

BJ^T/p53KD/p16KD/ST^ cells, lacking Ras^V12^, were retrovirally transduced to express the indicated transgenes or with an empty vector as a control. Cells were injected subcutaneously in immunocompromised NOD-*scid Il2rg*^*−*/*−*^ mice (Chen *et al*, 2009).

Tumors are shown in Supplementary Fig S2.

### Ras acts via SOCS5/6 to control YAP levels

**Figure 2:**
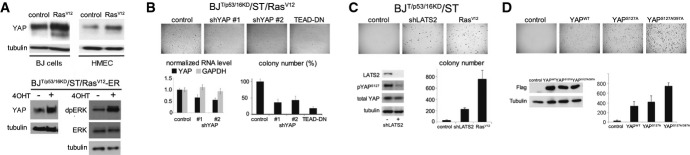
Regulation of YAP activity by Ras is required for cellular transformation Upper panels: effect of Ras^V12^ expression on YAP1. BJ and HMEC ^T/p53/p16KD^/ST cells were transduced to express Ras^V12^ or with empty vector control and cultured for 4 days. Blots were probed with anti-YAP1 and with anti-tubulin to control for loading. Lower panels: BJ^T/p53/p16KD^/ST cells were retrovirally transduced to express an estrogen receptor-Ras^V12^ fusion protein. Cells were treated with 10^−7^ M 4-hydroxytamoxifen (4OHT) to activate Ras^V12^. Right: anti-dpERK was used as a control for activation of ERK upon induction of ER-Ras^V12^.BJ^T/p53p16KD^/ST/Ras^V12^ cells were transduced to express two different shRNAs targeting YAP1, or a dominant-negative form of TEAD2 (TEAD-DN). Lower left: *YAP* mRNA level was measured by quantitative PCR to monitor the efficacy of the shRNAs. Data show the average of three independent experiments ± SD. *GAPDH* RNA was used as a control. Lower right: Colony number: average of three independent experiments, represented as % of empty vector control ± SD. *P* < 0.05 comparing sh-YAP#1 to control. *P* < 0.01 comparing sh-YAP#2 to control. *P* < 0.001 comparing TEAD-DN to control.BJ^T/p53p16KD^/ST cells were transduced to express an shRNA targeting LATS2. Ras^V12^ was used as a positive control. Empty vector served as a negative control. *LATS1* mRNA was undetectable in BJ and HMEC cells. Lower left: immunoblot for LATS2 protein to monitor the efficacy of the shRNA. The effect of LATS2 depletion on YAP activity was monitored by phospho-specific antibody to pS127 YAP. Anti-YAP and anti-tubulin were used to control for loading. Lower right: colony number: average of three independent experiments ± SD.BJ^T/p53p16KD^/ST cells were transduced to express Flag-tagged wild-type YAP and mutant forms of YAP insensitive to LATS kinases. Empty vector served as a negative control. Lower left: immunoblot for YAP protein. Anti-tubulin was used to control for loading. Lower right: Colony number: average of three independent experiments ± SD. Comparable amounts of the three YAP proteins were expressed. Note the increased potency of the LATS-insensitive forms of YAP. Source data are available online for this figure.

SOCS-box proteins can act as substrate recognition elements in oncogenic Cullin-RING-E3 ubiquitin ligase complexes (Linossi & Nicholson, 2012). The finding that Ras acts via SOCS5/6 prompted us to ask whether the SOCS-box proteins regulate expression of Ras targets linked to anchorage independent growth. Ras has been shown to regulate YAP via inactivation of LATS kinases (Reddy & Irvine, 2013). To ask whether regulation of YAP is involved in Ras-mediated anchorage independent growth, we first confirmed that expression of oncogenic Ras^V12^ increased total YAP1 protein expression in primary human cells (Fig2A). To assess the role of YAP in soft agar colony formation, two methods were used to lower YAP activity. First, shRNA-mediated depletion was used to lower YAP transcript levels (Fig2B, Supplementary Fig S4). Both shRNAs significantly reduced soft agar colony formation (Fig2B). Second, a dominant-negative form of TEAD (Liu *et al*, 2012; Liu-Chittenden *et al*, 2012; Johnson & Halder, 2014) was used to lower YAP (and TAZ) activity. TEAD-DN expression significantly reduced colony formation (Fig2B). Reciprocally, activation of endogenous YAP and TAZ by depletion of LATS2 was sufficient to bypass the requirement for Ras^V12^ to support anchorage independent growth *in vitro* and to support tumor formation in mouse xenograft assays (Fig2C, Table1, Supplementary Figs S3 and S5). Similar results were obtained by overexpressing YAP1 or LATS-insensitive versions of YAP1 (Fig2D, Table1; Supplementary Fig S6 shows HMEC). BJ cells proved to be relatively insensitive to native TAZ, but could be transformed by LATS-insensitive TAZ (Supplementary Fig S7). These experiments provide evidence that YAP activity is required for Ras-mediated transformation of primary human cells and for formation of xenograft tumors.

**Figure 3:**
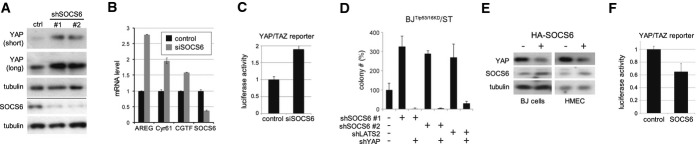
SOCS6 acts via YAP Effect of depleting SOCS5/6 on YAP1. BJ^T/p53/p16KD^/Ras^V12^/ST cells were transduced to express two independent shRNAs targeting SOCS6 or with empty vector. Blot probed with anti-YAP1. Anti-tubulin was used to control for loading. Below the line: Blot probed with anti-SOCS6 in a separate experiment.Quantitative real-time RT–PCR to measure YAP target levels in BJ^T/p53/p16KD^/ST cells. Cells were treated with siRNA to deplete SOCS6 or with a scrambled control siRNA. The efficacy of SOCS6 depletion is shown at right. Error bars: mean ± SD for three independent experiments. Increased expression of the YAP targets was significant comparing SOCS6-depleted and control cells (ANOVA: *P* < 0.0001).Luciferase reporter assay. The 8× GTIIC luciferase reporter, containing 8 TEAD binding sites (Dupont *et al*, 2011), was used to monitor YAP/TAZ activity in HEK cells. Cells were transfected with siRNA to deplete SOCS6 or with a control siRNA. Data were normalized to co-transfected Renilla luciferase to control for transfection efficiency and to the empty vector control. Histogram: average of three independent experiments ± SD. *P* < 0.01 comparing SOCS6-depleted and control cells.Effect of co-depleting SOCS5/6 and YAP1 on colony formation. BJ^T/p53/p16KD^/Ras^V12^/ST cells were transduced to express shRNAs targeting SOCS6 or LATS2 with or without shRNA targeting YAP1. Colony number: average of three independent experiments, represented as % of control ± SD. *P* < 0.01 comparing sh-SOCS6#1 or #2 to control, *P* < 0.001 comparing sh-SOCS6#1 or #2 with and without shYAP. *P* < 0.05 comparing shLATS to control and shLATS with shYAP.Effect of SOCS6 overexpression on YAP1. BJ^T/p53/p16KD^/Ras^V12^/ST and HMEC^T/p53/p16KD^/Ras^V12^/ST cells were transduced to express HA-tagged SOCS6 or with an empty vector. Blots probed with anti-YAP1 and anti-tubulin.Luciferase reporter assay. HEK cells were transfected to express HA-SOCS6 or with empty vector as a control. Data were normalized to co-transfected Renilla luciferase to control for transfection efficiency and to the empty vector control. Histogram: average of three independent experiments ± SD. *P* < 0.05 comparing SOCS6-overexpressing cells to the vector control. Source data are available online for this figure.

Ras activation reduced SOCS5/6 levels. We next asked whether reducing SOCS5/6 was sufficient to reproduce the effects of Ras^V12^ on YAP expression and activity. shRNA-mediated depletion of SOCS5/6 in primary BJ cells increased YAP1 protein levels (Fig3A) and increased expression of YAP target genes, notably Amphiregulin (*AREG*, Fig3B). YAP luciferase reporter activity was also increased (Fig3C). Increased YAP activity can support colony formation. Figure3D shows the effects of lowering YAP levels in cells activated by depletion of SOCS5/6 or LATS2. In both cases, depletion of YAP was sufficient to reduce colony formation induced by depletion of SOCS5/6 or LATS2 (Fig3D). We noted that YAP depletion was less effective in the LATS2 depleted cells, perhaps because of TAZ activation. Thus, YAP activation appears to be required to mediate the effects of SOCS6. Overexpression of HA-tagged SOCS6 had the opposite effect, reducing YAP1 protein levels in BJ and HMEC cells (Fig3E) and reducing YAP luciferase reporter activity (Fig3F).

In aggregate, these experiments provide evidence that activation of endogenous YAP, mediated via downregulation of the SOCS-box proteins, is an essential mediator of the activity of oncogenic Ras^V12^.

### SOCS6 promotes ubiquitylation of YAP1

**Figure 4:**
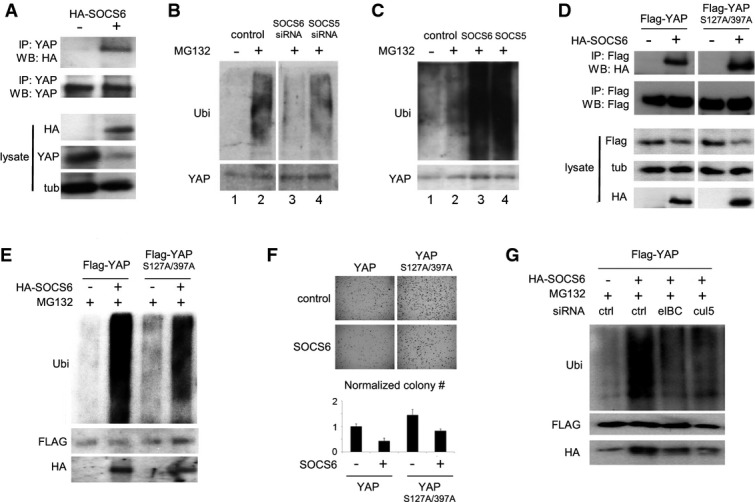
SOCS6 regulates YAP ubiquitylation Co-immunoprecipitation of SOCS6 with YAP1. HEK293T cells were transfected to express HA-tagged SOCS6 and treated with MG132 to block proteasome activity. Anti-YAP1 antibody was used for IP. Blots were probed with anti-HA to visualize HA-SOCS6. Total YAP1 was lower in cells expressing SOCS6. Co-transfection with ubiquitin increased recovery of SOCS6 in the YAP1 IP (not shown). 30% of the IP was loaded for immunoblot analysis. Lysate: 5% of the input.Effect of depleting SOCS5 and SOCS6 on YAP1 ubiquitylation. BJ cells were transfected with siRNAs to target SOCS5, SOCS6 or with a control siRNA. Cells were co-transfected with ubiquitin and treated with MG132 (+) or DMSO control (−) for 5 h before IP with anti-YAP1. Upper panel probed with anti-ubiquitin (Ubi). Lower panel probed with anti-YAP1. All samples were run on the same gel with an intervening lane removed (space between lanes 2 and 3).Effect of SOCS5 and SOCS6 overexpression on YAP1 ubiquitylation. Samples prepared as in (D).Co-IP of SOCS6 with phosphosite mutant YAP1. HEK293T cells were transfected to express HA-tagged SOCS6 and Flag-tagged YAP1 or YAP1^S127A/S397A^. Cells were treated with MG132. Anti-Flag antibody was used for IP. Blots were probed with anti-Flag to visualize recovered Flag YAP1 proteins and with anti-HA to visualize HA-SOCS6. All samples were run on the same gel with an intervening lane removed. 30% of the IP was loaded for immunoblot analysis.Effect of SOCS6 overexpression on YAP1 and YAP1^S127A/S397A^ ubiquitylation. Samples prepared as in (D).Effect of SOCS6 overexpression on colony formation in BJ^T/p53/p16KD^/ST cells expressing YAP1 or YAP1^S127A/S397A^. Colony number is the average of three independent experiment normalized to YAP1 control cells without SOCS6 overexpression ± SD. *P* < 0.05 comparing SOCS6-expressing cells to their respective controls.HEK293T cells were transfected with Flag-tagged YAP1, HA-tagged SOCS6 and with siRNAs targeting Elongin B/C or Cullin-5. Left panel: samples were immunoprecipitated with anti-Flag and probed with the indicated antibodies. SOCS6 overexpression increased ubiquitylation of Flag-tagged YAP1. This increase was limited by depletion of either Elongin B/C or Cullin-5. Endogenous SOCS6 was recovered at lower level in the Flag-IP sample without SOCS6 overexpression. Source data are available online for this figure.

As a first step toward testing the hypothesis that SOCS-box proteins act as substrate recognition elements for YAP1, we performed co-immunoprecipitation assays. Immunoprecipitation of HA-tagged SOCS6 pulled down endogenous YAP1 protein from HEK293T cells (Fig4A). Interaction between YAP1 and SOCS6 was readily detected even though SOCS6 expression reduced total YAP1 levels in these cells. To explore the effects of the SOCS proteins on YAP1 ubiquitylation, HEK293T cells were transfected with siRNAs targeting SOCS5 or SOCS6. Endogenous YAP1 was immunoprecipitated and the level of ubiquitylation monitored by immunoblotting. Co-depletion of SOCS5/6 using SOCS6 siRNA lowered the level of YAP1 ubiquitylation (Fig4B). Depletion of SOCS5 alone had a milder effect. Conversely, overexpression of SOCS5 or SOCS6 increased YAP1 ubiquitylation (Fig4C). Thus, interaction with the SOCS proteins targets YAP1 for ubiquitylation.

Phosphorylation of YAP1 by LATS1/2 targets YAP for βTrCP-SCF-mediated ubiquitylation and degradation (Zhao *et al*, 2010). Mutation of the LATS site S381 in mouse YAP was sufficient to prevent βTrCP-SCF mediated degradation of YAP1 (Zhao *et al*, 2010). Mutation of human YAP1 at S127 and S397 (corresponding to S381 in mouse) did not prevent interaction with SOCS6. Flag-YAP1 or Flag-YAP1^S127A/S397A^ proteins co-immunoprecipitated with HA-SOCS6 from HEK293T cells (Fig4D). SOCS6 expression increased ubiquitylation of YAP1^S127A/S397A^, albeit somewhat less efficiently than with the native YAP1 protein (Fig4E). As shown in Fig1C for Ras-transformed cells, SOCS6 expression also reduced soft agar growth of cells expressing native YAP or YAP1^S127A/S397A^ (Fig4F). Comparable results were obtained with the LATS-insensitive TAZ^S89A^ protein: overexpression of SOCS6 reduced TAZ^S89A^ levels and reduced soft agar growth (Supplementary Fig S7). The Cullin-RING-E3 ubiquitin ligase complexes, in which SOCS-box proteins act, are molecularly distinct from the βTrCP-SCF ubiquitin ligase complexes previously implicated in YAP ubiquitylation (Zhao *et al*, 2010). To assess the contribution of the Cullin-RING-E3 ubiquitin ligase complex in control of YAP activity, we used siRNAs to deplete the Elongin B/C subunits and Cullin-5. Depletion of these proteins blunted the ability of overexpressed SOCS6 to increase YAP ubiquitylation (Fig4G). Together, these findings provide evidence that SOCS6 acts with a Cullin-5-Elongin B/C ubiquitin ligase complex to control YAP protein turnover. The SOCS6-containing ubiquitin ligase complex does not require the LATS-primed motif that regulates YAP turnover by βTrCP-SCF. Thus, there appear to be two molecularly distinct mechanisms by which YAP is targeted for degradation.

### SOCS6 in cancer

**Figure 5:**
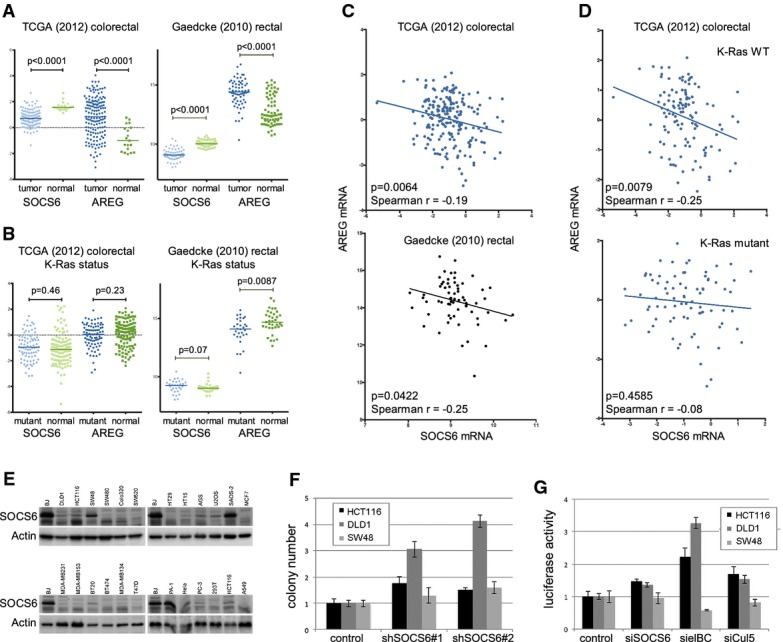
SOCS6 activity in colorectal cancer Scatter plots comparing *SOCS6* and *AREG* mRNA levels in tumor and normal control samples from the TCGA colorectal cancer dataset (Network, 2012) and the Gaedcke rectal carcinoma dataset (Gaedcke *et al*, 2010). Median, upper, and lower quartiles are indicated. *P*-values were determined using the Mann–Whitney test (two-tailed).Scatter plots comparing *SOCS6* and *AREG* mRNA levels in tumors with mutated versus normal K-Ras. *P*-values were determined using the Mann–Whitney test (two-tailed).Scatter plots comparing *SOCS6* and *AREG* mRNA levels in individual tumors from the TCGA and Gaedcke datasets. Spearman's rho and *P*-value are shown in the figures.Scatter plots comparing *SOCS6* and *AREG* mRNA levels in individual tumors from the TCGA dataset separated by K-Ras status.Immunoblots of a panel of cancer cell lines probed with anti-SOCS6 and anti-actin.Soft agar colony formation by three colorectal cancer cell lines transduced with shRNAs to deplete SOCS6 or with empty vector as a control. Data represent the average of three independent experiments ± SD. HCT116 and DLD1 cells *P* < 0.05 compared to control for each siRNA. SW48 cells: not significant.YAP/TAZ luciferase reporter assay in colorectal cancer cell lines transduced with shRNAs to deplete SOCS6, Elongin B/C, Cullin-5 or with empty vector as a control. Data represent the average of three independent experiments ± SD. HCT116 and DLD1 cells *P* < 0.05 compared to control for each siRNA. SW48 cells: not significant. Source data are available online for this figure.

Given the evidence that Ras^V12^ downregulates SOCS6 and that SOCS6 depletion is required for efficient Ras^V12^-mediated cellular transformation, we sought to explore the cancer relevance of SOCS6. The *SOCS6* gene is located in an interval frequently deleted in colorectal cancer (Fearon *et al*, 1990). *SOCS6* mRNA levels were significantly reduced in a variety of cancers (http://www.oncomine.org), most notably colorectal carcinomas. *SOCS6* mRNA was significantly reduced in tumors versus normal tissue in the TCGA colorectal carcinoma dataset (TCGA Network, 2012). *SOCS6* mRNA was significantly reduced in a second dataset (Gaedcke *et al*, 2010), which contains 65 rectal adenocarcinomas and 65 controls from adjacent normal tissue (Fig5A), as well as other colorectal cancer datasets (Supplementary Table S8). Interestingly, the YAP target Amphiregulin (*AREG*, Zhang *et al*, 2009) was significantly increased in tumor samples from both datasets compared to the controls (Fig5A). At the level of individual tumors, we observed a significant inverse correlation between *SOCS6* and *AREG* mRNA levels (Fig5C). Significant inverse correlation was also found between *SOSCS6* and *AREG* levels in skin cutaneous melanoma and glioblastoma multiforme (Supplementary Table S9). These observations are consistent with the hypothesis that lowering SOCS6 levels increases YAP activity in these tumors. Activating mutations of K-Ras occur in ˜40% of colorectal carcinomas (TCGA Network, 2012). To ask whether K-Ras status is correlated with *SOCS6* and *AREG* levels, we compared 80 tumors with mutant K-Ras versus 116 tumors with wild-type K-Ras from the TCGA dataset. There was no significant difference comparing *SOCS6* or *AREG* mRNA levels between these two groups (Fig5B). Interestingly, the inverse correlation between *SOCS6* and *AREG* mRNAs was observed in the tumors with wild-type K-Ras (*P* = 0.0079) but not in the K-Ras mutant tumors (*P* = 0.459, Fig5D). Indeed, the correlation was slightly stronger in the K-Ras wild-type tumors compared to the whole population (Spearman *r* = −0.25 versus *r* = −0.19). This correlation suggests that the impact of SOCS6 on YAP activity may be more important in the population of tumors where Ras pathway activity can be modulated by extracellular signals, than in the population that develop with constitutive high-level Ras activation.

Immunoblotting showed that SOCS6 protein levels were low in a variety of colorectal, breast, and other cancer cell lines, compared to primary human BJ cells (Fig5E). This prompted us to ask whether SOCS-mediated regulation of YAP activity might have been lost during the process of selection for growth of the tumor-derived cells in culture. Depletion of SOCS6 increased the ability of DLD1 and HCT116 colorectal cancer cells to form colonies in soft agar, but had limited impact on colony formation by SW48 cells (Fig5F, Supplementary Fig S10). As in BJ cells, SOCS6 depletion led to an increase in YAP/TAZ activity in the luciferase reporter assay for DLD1 and HCT116 cells, but not for SW48 cells (Fig5G, Supplementary Fig S10). We note that the magnitude of the effect of SOCS6 depletion was less in the cancer cell lines than in BJ cells, perhaps reflecting the lower starting level of SOCS6 protein in these cells. Depletion of Elongin B/C or Cullin-5 increased YAP/TAZ reporter activity in DLD1 and HCT116 cells, but not in SW48 (Fig5G). These data provide evidence that SOCS6 and the Cullin-RING-E3 ubiquitin ligase mechanism for YAP/TAZ regulation has remained intact in some cell lines, but not in others. When this mechanism is intact, the level of SOCS6 activity influences anchorage independent growth.

### Positive feedback via YAP1 on the endogenous EGFR/Ras pathway

**Figure 6:**
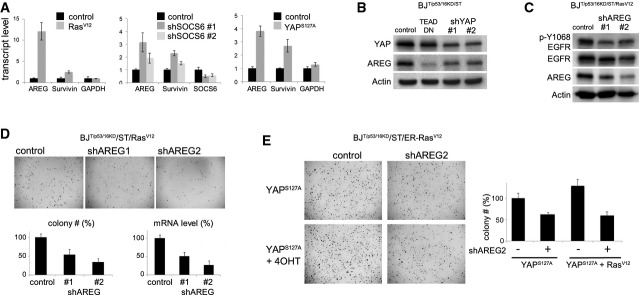
The Ras/SOCS/YAP/AREG feedback loop Upregulation of AREG and survivin in BJ^T/p53KD/p16KD/ST^ cells transduced to express Ras^V12^, two independent shRNAs targeting SOCS5/6, or YAP^S127A^. RNA was isolated from a subset of the cells prepared for the colony formation assay in Fig3A. The immunoblot control for SOCS6 depletion is shown in Fig3A. Transcript levels were measured by quantitative PCR. Data: average of three independent experiments ± SD. *GAPDH* was used as a control.Immunoblot showing the effect of YAP depletion on AREG protein levels. BJ^T/p53KD/p16KD/ST^ cells were transduced to express two independent shRNAs targeting YAP or TEAD-DN. Blots were probed with antibody to YAP and AREG. Actin served as a loading control.Immunoblot showing the effect of AREG depletion on EGFR activity in BJ^T/p53KD/p16KD/ST/Rasv12^ cells. Cells were transduced to express two independent shRNAs targeting AREG. Blots were probed with antibody to Y1068 phosphorylated EGFR to monitor EGFR activity, and with antibody to EGFR and AREG. Actin served as a loading control.Effect of AREG depletion on soft agar colony formation by fully transformation-competent BJ^T/p53KD/p16KD/ST^/Ras^V12^ cells. Cells were transduced to express two independent shRNAs targeting AREG or with empty vector as a control. Lower left: average colony number from three independent experiments ± SD shown as % of vector control. *P* < 0.05 comparing each siRNA to control. Lower right: *AREG* mRNA levels were measured by quantitative PCR to monitor shRNA efficacy.Effect of AREG depletion on soft agar colony formation by YAP1^S127A^-expressing cells. BJ^T/p53KD/p16KD/ST^/ER-Ras^v12^ cells were transduced to express YAP1^S127A^ and with shRNA targeting AREG or with empty vector as a control. Below: average colony number from three independent experiments ± SD, normalized to YAP1^S127A^-expressing cells. *P* < 0.05 comparing each siRNA to it respective control. Source data are available online for this figure.

The inverse correlation between *SOCS6* and *AREG* mRNA levels in human cancers was intriguing. In primary cells, shRNA-mediated depletion of SOCS6 increased *AREG* mRNA levels, as did expression of YAP or Ras^V12^ (Fig6A). Reciprocally, shRNA-mediated depletion of YAP, or expression of the TEAD2 dominant-negative protein in these primary cells, reduced AREG expression (Fig6B). AREG has been shown to act via EGFR to mediate YAP activity in MCF10A cells (Zhang *et al*, 2009). Using a phospho-specific antibody that recognizes activated EGFR, we confirmed that depletion of AREG reduced EGFR activation in our primary cell model (Fig6C).

To ask whether induction of AREG contributes to Ras-mediated transformation, we depleted AREG using two independent shRNAs and measured soft agar growth. Ras^V12^-dependent colony formation was significantly reduced by both shRNAs in BJ cells (Fig6D, *P* < 0.05). AREG depletion also significantly reduced YAP1^S127A^ mediated colony formation, and this was not rescued by co-activation of Ras^V12^ (Fig6E, *P* < 0.05). These findings suggest that YAP1-dependent induction of AREG is required for efficient Ras^V12^-driven transformation. To test whether the requirement for AREG is mediated through activation of EGFR, we treated with monoclonal antibodies to EGFR, and observed that this significantly reduced the efficiency of YAP-mediated soft agar colony formation (Supplementary Fig S11). These observations provide evidence that AREG-mediated activation of endogenous EGFR is required for full transformation downstream of YAP.

## Discussion

### Opposing activities of Ras and Hippo pathways converge on regulation of YAP turnover

Regulation of protein turnover by ubiquitin-mediated protein degradation has long been recognized as an important mechanism for regulating the activity of signal transduction pathways in cancer (Marmor & Yarden, 2004; Fulda *et al*, 2012). Proteins involved in ubiquitin-mediated protein degradation have been identified as tumor suppressors (reviewed in (Wang *et al*, 2014). For example, FBxw7/Archipelago, a component of the SCF ubiquitin ligase complex that target cyclinE for degradation is mutated or inactivated in a variety of cancers (Moberg *et al*, 2001; Strohmaier *et al*, 2001; Mao *et al*, 2004; Iwatsuki *et al*, 2010).

The SCF βTrCP complex contributes to the tumor suppressor activity of the Hippo pathway by targeting LATS-phosphorylated YAP/TAZ for degradation (Liu *et al*, 2010; Zhao *et al*, 2010). We have presented evidence that Ras acts via a SOCS5/6 to control YAP turnover. SOCS6 serves as a recognition factor for YAP and promotes its degradation via an Elongin B/C Cullin-5 ubiquitin ligase complex. Downregulation of SOCS5/6 by Ras promotes YAP stability. Abrogation of SOCS6-mediated YAP turnover is required for efficient Ras-mediated cellular transformation. Thus, YAP is a convergence point for the opposing activities of the Hippo and Ras pathways, which act via distinct ubiquitylation mechanisms to control YAP turnover.

### Balancing Ras-driven proliferation and Hippo tumor suppressor activity

The EGFR/Ras and Hippo pathways are central to growth control during normal animal development, in addition to their roles in disease. Multiple links between these pathways have been reported. EGFR and the Ras/MAPK pathway act via phosphorylation of Ajuba proteins to inactivate LATS kinases (Reddy & Irvine, 2013). By this route, Ras leads to reduced YAP phosphorylation and increased YAP activity. Thus, EGFR/Ras can promote YAP activity by two independent mechanisms: inhibition of LATS kinases reduces SCF βTrCP dependent YAP turnover and inhibition of SOCS5/6 expression reduces Elongin B/C/Cullin-5-dependent YAP turnover. It is noteworthy that both mechanisms impact YAP stability and that they use different mechanisms to control YAP ubiquitylation.

Interestingly, Ras has also been reported to promote Hippo pathway activity: oncogenic K-Ras can activate MST2 kinase (Matallanas *et al*, 2011). Activation of MST/Hippo kinases leads to increased YAP phosphorylation and turnover, lowering YAP activity. Thus, oncogenic forms of Ras seem to promote the tumor suppressor activity of the Hippo pathway, while at the same time opposing Hippo pathway activity by increasing YAP activity.

Why should Ras act both positively and negatively on Hippo pathway activity? Cells need some level of YAP activity to proliferate. Hippo pathway activity limits YAP activity. This brake provides an essential tumor suppressor activity. Proliferative cues from Ras/MAPK pathway activation may alleviate Hippo activity thereby increasing YAP activity to support proliferation. Having both positive and negative links between Ras and Hippo provides the opportunity for input from other pathways to adjust the balance between proliferative and antiproliferative states. Indeed YAP activity can also be regulated by other pathways including Wnt, Notch, and TGF-β, reflecting the central importance of YAP in proliferation (Barry & Camargo, 2013). During selection for neoplastic growth driven by oncogenic Ras^V12^, genetic or epigenetic alterations may shift the balance between different homeostatic mechanisms. Ultimately, for cells that go on to form colonies in soft agar, this balance shifts in favor of the YAP-stabilizing output of the Ras^V12^/SOCS/YAP mechanism. We suggest that this is also likely to be the case for tumors expressing oncogenic Ras mutations *in vivo*.

### A Ras/SOCS/YAP positive feedback loop

Oncogenic Ras is required for anchorage independent growth of primary human cells (Hahn *et al*, 1999, 2002). Our findings show that the requirement for oncogenic Ras^V12^ can be replaced by providing sufficient YAP activity. This can be accomplished by depletion of LATS2, to reduce the impact of the Hippo pathway on endogenous YAP/TAZ, or by overexpression of YAP1. Stabilization of endogenous YAP, through downregulation of SOCS5/6 is also sufficient to transform primary cells without expression of oncogenic Ras^V12^.

By controlling SOCS5/6 expression levels, Ras regulates YAP stability through ubiquitin-mediated proteasome degradation. YAP in turn acts via AREG to activate the endogenous EGFR pathway, presumably leading to activation of other effector pathways, including those regulated by endogenous Ras: MAPK, PI3K, and RalGEF. Feedback via the EGFR/Ras pathway would lead to increased YAP stability, creating a positive feedback loop. We have provided evidence that the potency of the Ras^V12^ and YAP1 oncogenes depends on this mutually reinforcing feedback loop.

These connections may help to explain the potency of activated Ras^V12^ and YAP in transformation. Cells expressing oncogenic forms of activated H-Ras and K-Ras remain sensitive to activation of the endogenous EGFR/Ras pathway (Young *et al*, 2013). PI3K can serve as an effector of activated K-Ras in pancreatic cancer cells (Prahallad *et al*, 2012; Eser *et al*, 2013) and K-Ras activity can modulate EGFR activation in colorectal cancer cells (Prahallad *et al*, 2012). It has been known that YAP can feedback on the EGFR/Ras pathway via induction of AREG (Zhang *et al*, 2009). Our findings show that oncogenic Ras acts via controlling YAP1 turnover, to activate the endogenous EGFR/Ras pathway and that this Ras/YAP1/AREG feedback loop is important for Ras-mediated transformation.

### Ras and YAP in cancer

Activating mutations affecting K-Ras, N-Ras, or B-RAF have been found in 55% of sequenced colorectal cancers, suggesting that activation of the Ras/MAPK pathway plays a key role (TCGA Network, 2012). In light of the link between oncogenic Ras and YAP1 levels, it is noteworthy that elevated YAP and TAZ protein levels was recently linked to poor prognosis for CRC patients (Wang *et al*, 2013). We observed a significant correlation between low SOCS6 levels and elevated *AREG* mRNA levels in CRC. Perhaps activation of the endogenous EGFR/ErbB pathway via the SOCS6/YAP1/AREG feedback loop contributes to sustaining proliferation and resistance to apoptosis. Recent reports have suggested that low SOCS6 levels are associated with poor prognosis in hepatocellular carcinoma and prostate cancer (Qiu *et al*, 2013; Zhu *et al*, 2013). Monoclonal antibodies to EGFR are in clinical use to treat a subset of CRC patients. Evidence is accumulating that patients with elevated EGFR expression and overexpression of EGFR ligands show sensitivity to anti-EGFR, whereas those with mutations in RAS, RAF, and PTEN may not (Di Fiore *et al*, 2010). It will be of interest to explore whether CRC patients with low SOCS6 levels show elevated YAP/AREG/EGFR pathway feedback activity. Our findings suggest that this pathway may be of more relevance in cases with a normal K-Ras gene.

Our findings provide evidence that expression of YAP is sufficient to bypass the requirement for Ras activity in transformation of primary human cells. While this manuscript was under review, two groups reported that YAP expression could bypass the requirement for Ras in other tumor models. In one study, YAP expression was found to support growth of a K-Ras dependent colon cancer cell line in the absence of K-Ras expression (Shao *et al*, 2014). The other study found that the YAP locus was amplified in K-Ras induced pancreatic tumors that were able to regrow following removal of K-Ras expression (Kapoor *et al*, 2014).

The findings reported here provide a molecular framework for understanding the relationship between the Ras pathway and YAP activity, though Ras-mediated regulation of SOCS5/6 expression and the resultant effects on YAP protein turnover. Analysis of colorectal cancer data sets suggests that this regulatory relationship may be functionally relevant in tumors with a normally functioning Ras pathway. We have provided evidence for a mechanistic relationship between SOCS6, YAP, and the YAP target AREG that contributes to Ras-mediated transformation of primary cells. This relationship appears to be preserved in colorectal tumors with wild-type K-Ras, but not in those with activating mutations in K-Ras. It will be of interest to explore whether subsets of tumors with defined molecular classification show a stronger association.

## Materials and Methods

### shRNA primers and plasmid construction

shRNAs were cloned into pRetrosuper-Blast (Voorhoeve *et al*, 2006) in which the Blast ORF was fused to YFP. SOCS6 shRNA #1: GCTGAAAGTATGCGCTGTCAT #2: AATTTTCAGCTACACACCT; YAP1 shRNA #1: CCAGAGAATCAGTCAGAGT #2: TGTATTGCTGACCTCTTTC; AREG shRNA #1: CACTGCCAAGTCATAGCCATAC #2: GAACGAAAGAAACTTCGACAAC (Zhang *et al*, 2009); lats2 shRNA #1: CAAGCATCCTGAGCACGCA #2: CTCTGTGACTGGTGGAGTG.

### Mammalian cell culture

Human primary BJ fibroblasts were cultured in DMEM with 10% FBS. Human mammary epithelial cells were cultured in MEGM mammary epithelial medium (Lonza CC-3151). BJ and HMEC cells were first retrovirally transduced to express ecotropic receptor and then modified with retrovirally transduced p53 shRNA, p16^INK4A^ shRNA, small T antigen, inducible ER-Ras^V12^, and hTert as described (Elenbaas *et al*, 2001; Voorhoeve & Agami, 2003). Retrovirus was made by calcium phosphate transfection of 293T cells and harvested after 48 h. BJ or HMEC cells were transduced with virus for 24 h and selected in medium containing 2–4 μg/ml blasticidin for 2–4 days before plating in soft agar.

### Luciferase reporter assays

To measure YAP activity, we used the 8xGTIIC YAP/TAZ firefly luciferase reporter (Dupont *et al*, 2011). Cells were transfected to express the indicated proteins and with Renilla luciferase as a control for signal normalization. Dual luciferase assays were performed according to the manufacturers protocol (Promega). Three independent transfections were carried out for each experiment. Data were normalized to the empty vector control and presented as average ± SD.

### Immunoprecipitation

HEK29T cells grown in 10-cm dish were transfected with pcDNA3.1-HA-SOCS6, pcDNA3-c-myc-ubiquitin, and native or mutant forms of YAP1 using calcium phosphate. Cells were harvested 48 h post-transfection. After trypsinization, the cell pellet was washed with chilled PBS and resuspended in 0.8 ml lysis buffer (20 mM HEPES at pH 7.9, 200 mM KCl, 2% complete protease inhibitor cocktail (Roche, #11836153001), 20% glycerol, 0.5% NP-40). Cells were lysed by passage through a 27^1/2^G needle 5–10 times. The lysate was incubated on ice for 10 min. Nuclei were removed by centrifugation at 10,000 *g* for 10 min at 4°C. The supernatant was passed through a 0.45-μm syringe filter (Sigma, #Z227463). Rabbit polyclonal anti-Flag (Sigma, 1:200 dilution) or rabbit polyclonal anti-YAP (Santa Cruz, 1:100) were used in the IP, as described (Hong *et al*, 2009).

### Ubiquitylation assays

For siRNA experiments, HEK293T cells were transfected ± SOCS5/6 siRNA in 10-cm dishes (Dharmafect). For SOCS overexpression, HEK293T cells were calcium phosphate transfected with pcDNA-SOCS5/6 or with empty vector and pcDNA-c-myc-ubiquitin. After transfection, cells were treated with MG132 (20 μg/ml) for 5 h before IP with rabbit polyclonal anti-YAP (Santa Cruz, 1:100). Ubiquitylation was assessed using rabbit polyclonal anti-ubiquitin (Sigma, 1:1,000). Cells treated with an equal volume of DMSO without MG132 were used as control for ubiquitin detection**.**

### Soft agar assay

Cells were resuspended in DMEM containing 0.4% low-melting agarose (Sigma, type VII) and 10% FBS. Two ml of 1% soft agarose was layered and solidified at room temperature in 6-well plates before addition of the cell-agar mixture. Freshly plated cells were incubated at 4°C for 15–20 min. One ml of culture medium was added to each well. Colony formation was assayed after 2 weeks. Colonies were stained and photographed under identical settings and counted using MATLAB.

### Xenograft assay

Animal work was carried out according to guidelines approved by A*STAR Biological Resource Center and Institutional Animal Care and Use committee (IACUC No: 120768) in Singapore. 4–5 week NOD-*scid Il2rg*^*−/−*^ mice (Chen *et al*, 2009) were prepared for injection. Two million cells were injected subcutaneously into each flank adjacent to each of the legs. Tumors were harvested when they reached 2 cm or at 9 weeks.

### Real-time quantitative RT–PCR

RNA was extracted by lysing cells in TRIzol. Purified RNAs were treated with DNase I. Oligo-dT based reverse transcription was performed using Superscript III. Real-time quantitative PCR was performed using Sybr Green reagents on an ABI 7500 fast real-time PCR platform.
