# Supplementary material for: Opposing activities of the Ras and Hippo pathways converge on regulation of YAP protein turnover
Source: EMBO J. 2014 Sep 1;33(21):2447–57. doi: 10.15252/embj.201489385 (PMC4283404; doi:10.15252/embj.201489385)
Supplement: Supplementary file 1 [file embj0033-2447-sd1.pdf]

THE
EMBO
JOURNAL

Manuscript EMBO-2014-89385

Opposing activities of the Ras and Hippo pathways converge on regulation of YAP protein turnover

Xin Hong, Hung Thanh Nguyen, Qingfeng Chen, Rui Zhang, Zandra Hagman, P. Mathijs Voorhoeve and Stephen M Cohen

Corresponding author: Stephen Cohen, Institute for Molecular Cell Biology

Review timeline:

Presubmission Enquiry:	22 April 2014
Editorial Correspondence:	22 April 2014
Submission date:	26 June 2014
Editorial Decision:	14 July 2014
Revision received:	10 August 2014
Accepted:	15 August 2014

Transaction Report:

(Note: With the exception of the correction of typographical or spelling errors that could be a source of ambiguity, letters and reports are not edited. The original formatting of letters and referee reports may not be reflected in this compilation.)

Editor: Thomas Schwarz-Romond

Presubmission Enquiry 22 April 2014

I'm writing to ask your view on whether EMBO J might be interested in considering the following manuscript:

"Opposing activity of oncogenic Ras and the Hippo tumor suppressor pathway converge on regulation of YAP protein turnover"

YAP is the key target through with the Hippo tumor suppressor pathway regulates cell growth. YAP overexpression (or activation) is sufficient to produce tumors, in certain contexts. YAP is phosphorylated by LATS kinase (Hippo pathway) with two outcomes: reduced activity due to retention in the cytoplasm and increased YAP protein turnover mediated by the bTRCP-SCF ubiquitin ligase complex. So, Hippo works as a tumor suppressor by keeping YAP activity low (similar with the related protein TAZ).

Our work identifies oncogenic Ras as an independent regulator of YAP protein turnover, which acts in the opposite direction: stabilizing YAP.

· Ras activity in cellular transformation depends on its ability to stabilize YAP.

- Stabilizing YAP by other means bypasses the requirement for oncogenic Ras in cellular transformation and formation of xenograft tumors

· Ras acts on YAP stability independent of the Hippo pathway, via a different ubiquitin ligase complex.

· We provide a mechanism of action for Ras via regulation of SOCS6:

- SOCS6 recruits YAP for ubiquitylation by a complex including ElonginB/C and Cullin5.
- Regulation of SOCS6 is required for Ras-mediated cellular transformation.
- SOCS6 expression can block the transforming action of oncogenic Ras
- Depletion of SOCS6 is sufficient to replace oncogenic Ras in anchorage independent growth and in formation of xenograft tumors.

· Cancer relevance: SOCS6 levels are low in many cancers. In colorectal cancers with low SOCS6, YAP activity is high, and a critical YAP target, AREG, is upregulated.

· AREG is a ligand for EGF receptors. Upregulation of AREG is required for efficient Ras mediated transformation.

We are tidying up a few experiments and expect to have the manuscript ready for submission in about a month. I take the opportunity to ask whether you think EMBO J would like to review this work.

Editorial Correspondence 22 April 2014

Thank you for considering The EMBO Journal.

This sounds like a very complete dataset and while this field is fast-moving, I would be delighted to discuss the paper with my colleagues in anticipation that it would be send out for peer-assessment.

1st Editorial Decision 14 July 2014

Thank you very much for submitting your study on Ras-mediated control of YAP-turnover that mechanistically informs the oncogenic Ras interaction with Hippo's tumor suppressor function for consideration to The EMBO Journal editorial office.

I received comments from two expert scientists that assessed suitability of your submission for The EMBO Journal having be alerted about the recently published studies in an alternative title and EMBO's position to complementary studies arising in different laboratories.

From these comments, principal interest in timely publication of your results becomes obvious. However, both referees also point to missing crucial controls, demand a certain level of necessary, further validation and possible extension to the proposed EGFR-signal integration/further reaching positive feedback regulation.

From an editorial perspective, I judge

(i) the corroboration of the molecular findings
(ii) presentation of protein-level upon gene-depletion approaches,
(iii) stronger evidence for the new, molecular interrelations in Ras-driven tumors
(iv) strengthening the feed-forward regulation as most relevant for potential rapid pursuit.

Inclusion of possibly already available data as to expand to various Ras-alleles, additional YAP-targets would address (i). (ii) are essential controls to increase confidence in the molecular changes and need to be added. For point (iii), I am convinced that the mouse-based data from the complementary papers support some of your results. This should be constructively incorporated into a revised version. I would further suggest exploitation of existing patient-derived datasets to consolidate the proposed molecular interplay in a tumor-context.

Conditioned on the rationale and constructive comments of our referees, I would be prepared to invite a revised version of your manuscript for final assessment. I realize that these request are in part challenging, particularly as we would have to work strictly to a three month (preferably faster!) deadline.

Please do not hesitate to get in touch with regard to feasibility and anticipated timeline for the necessary revisions (due to time constrains preferably via e-mail).

I also have to formally remind you that The EMBO Journal only considers one major round of revisions and look forward to hear from you/receive a suitably revised version to your earliest convenience!

REFEREE REPORTS:

Referee #1:

In this manuscript, Hong et al. follow up on a prior study in which they showed that the Drosophila SOCS-box protein, Socs36E, potentiates EGFR signaling and transformation. They show that expression of oncogenic RAS suppresses SOCS5 and SOCS6 expression by 50% and that suppression of SOCS5 and SOCS6 substituted for RAS expression to transform human cells expressing SV40 ST and immortalized by the expression of hTERT and suppression of p16 and p53. Expression of SOSC6 suppressed RAS induced transformation. They speculated that SOCS5 and SOCS6 act as ubiquitin ligases to regulate the levels of YAP1 and showed that suppressing YAP1 expression or expression of a dominant inferring allele of TEAD reduced RAS induced transformation. They further showed in 293T cells that exogenously expressed SOCS6 was found in YAP1 immune complexes and that manipulating SOCS5 and SOCS6 levels affected the ubiquitination of YAP1. They then searched tumor expression data and found that tumors that express lower levels of SOCS6 showed increased AREG levels. They speculated that by regulating YAP1 levels, RAS induces the expression of AREG and show that suppressing AREG affects RAS induced tumor formation.

These studies are timely and of potential interest to investigators in several fields. The authors present a sweeping model for the relationship of RAS, SOCS5/6 and feedback regulation of AREG through YAP1. Although the data presented in this manuscript is consistent with this model, some of the connections require further evidence as the data could support several alternative explanations.

Major comments:

1. The authors argue that RAS regulates Hippo signaling to induce this feedback loop. Although YAP1 does function as a key effector of Hippo signaling, YAP1 also functions to regulate many transcription factors depending on the context. The data presented in this manuscript is most consistent with a role for YAP1 in KRAS induced transformation but do not definitively demonstrate that Hippo signaling is involved. Although they manipulate LATS to artificially regulate YAP1, they also demonstrate that the primary LATS phosphorylation sites are not those that regulate YAP1 stability in this context. Thus, although it remains possible that Hippo signaling may modify YAP1 to affect the pathway studied herein, there is insufficient evidence to claim that Hippo signaling is involved. As such, the authors need to modify their assertion throughout the manuscript that RAS regulates Hippo signaling in this context.

2. The authors extrapolate from engineered human cell lines and 293T cells to conclude that the observations presented herein are relevant to human tumors. This may well be the case but at a minimum, the authors should demonstrate that these relationships occur in tumors that have mutant RAS. Moreover, the authors use different cell lines for the transformation experiments than are used for biochemical experiments (ubiquitination, protein-protein interaction studies) and most of the biochemical studies involve overexpressed proteins. To provide strong evidence that these pathways are truly operating in RAS transformed cells, the authors should use some of the same cell lines throughout the manuscript.

3. The authors assume that AREG is the key YAP1 regulated gene involved in this feedback loop. Based on what is presented, it is unclear how the authors came to this conclusion. How many other genes show a reciprocal relationship with SOCS6 levels? Was AREG the only one? Furthermore, the authors have not demonstrated that YAP1 regulates AREG in this context nor have they shown that YAP1 and TEAD are present at the AREG gene. Although the data is consistent with this interpretation, it is impossible to know if this relationship is truly relevant and connected to changes in AREG levels.

4. Related to point #3, the authors focus on glioblastoma and colon cancers, where they argue that EGFR signaling is important for the pathogenesis of these cancers. However, this manuscript is focused on RAS. Is there a correlation between SOCS6 levels and AREG in tumors that have mutant RAS? Is there a relationship between mutant KRAS and AREG?

5. The authors never specify which RAS allele they are using. Based on citations, one might assume that they have used HRAS but this is never stated. Are the same findings true if one uses KRAS or NRAS?

6. The authors argue that a RAS-SOCS5/6-YAP1-AREG loop is critical for RAS induced transformation. How does RAS regulate SOCS5/6? Are the well studied RAS effector pathways (MEK, PI3K, RAL) important for this regulation?

7. In general, the studies demonstrating ubiquitination of YAP1 by SOCS5/6 are incomplete. What form of ubiquitination is regulated by SOCS5/6? What sites in YAP1 are critical for this modification. Is phosphorylation necessary to allow ubiquitination? Without this information, the reader is left wondering how direct the effect of SOCS5/6 are on YAP1.

Minor points

1. The introduction is written quite bluntly and reads as a series of declarative statements.

2. Figure 4C is of poor quality

3. Figure 6B. The p value seems to overstate the relationship shown. Have the authors accounted for multiple hypothesis testing?

4. The interaction of SOCS5 and SOCS6 is interesting but underdeveloped? Do these proteins form a complex that is required for ubiquitination?

Referee #2:

Hong et al have described a novel mechanism by which the oncogenic Ras promotes stabilization of the Hippo pathway mediator YAP1. They find that activated Ras opposes the Hippo-driven destruction of YAP1 by regulating the ubiquitin ligase complex substrate recognition factors SOCS5/6, which bind YAP1 to promote its ubuitination. The results are supported by a variety of overexpression and RNAi studies in cultured fibroblasts, and are extended to xenografts, and several human cancer cell lines.

Overall, the findings are of topical interest, especially in light of 2 recent related publications in Cell describing the role of YAP1 in bypassing a requirement for activated KRAS in maintaining malignant transformation. Here, the authors essentially build on that novel Ras-YAP1 link with an additional mechanistic aspect.

There is, however, a notable lack of somewhat standard controls throughout this manuscript. In particular, most of the shRNA and many of the overexpression/ectopic expression studies do not include protein blotting data to support the expected effects on expression of YAP1, Ras, TEAD, AREG, LATS2, and SOCS5/6. In a few cases, RNA levels are shown, but it's protein that really matters.

Which isoform of Ras is being used here? Probably H-Ras, since there is transforming activity in fibroblasts (which Kras largely lacks). Of course, Kras is the more relevant human oncogene.

The link to EGFR is weak, and the claim in the abstract that Rasv12 "activates the endogenous EGFR pathway" should be removed. The authors' inference that this pathway is involved (last paragraph of Results) is not supported by anything other than effects on ampiregulin expression, and the AREG shRNA study (which lacks protein expression controls). In fact, this is readily testable with EGFR kinase inhibitors. And, in my view, this is unlikely to work, especially when considering that EGFR inhibitory drugs have not proven to be clinically effective in Ras mutant cancers.

First Revision 10 August 2014

Ref 1

In this manuscript, Hong et al. follow up on a prior study in which they showed that the Drosophila SOCS-box protein, Socs36E, potentiates EGFR signaling and transformation. They show that expression of oncogenic RAS suppresses SOCS5 and SOCS6 expression by 50% and that suppression of SOCS5 and SOCS6 substituted for RAS expression to transform human cells expressing SV40 ST and immortalized by the expression of hTERT and suppression of p16 and p53. Expression of SOSC6 suppressed RAS induced transformation. They speculated that SOCS5 and SOCS6 act as ubiquitin ligases to regulate the levels of YAP1 and showed that suppressing YAP1 expression or expression of a dominant inferring allele of TEAD reduced RAS induced transformation. They further showed in 293T cells that exogenously expressed SOCS6 was found in YAP1 immune complexes and that manipulating SOCS5 and SOCS6 levels affected the ubiquitination of YAP1. They then searched tumor expression data and found that tumors that express lower levels of SOCS6 showed increased AREG levels. They speculated that by regulating YAP1 levels, RAS induces the expression of AREG and show that suppressing AREG affects RAS induced tumor formation.

These studies are timely and of potential interest to investigators in several fields. The authors present a sweeping model for the relationship of RAS, SOCS5/6 and feedback regulation of AREG through YAP1. Although the data presented in this manuscript is consistent with this model, some of the connections require further evidence as the data could support several alternative explanations.

Major comments:

1. The authors argue that RAS regulates Hippo signaling to induce this feedback loop. Although YAP1 does function as a key effector of Hippo signaling, YAP1 also functions to regulate many transcription factors depending on the context. The data presented in this manuscript is most consistent with a role for YAP1 in KRAS induced transformation but do not definitively demonstrate that Hippo signaling is involved. Although they manipulate LATS to artificially regulate YAP1, they also demonstrate that the primary LATS phosphorylation sites are not those that regulate YAP1 stability in this context. Thus, although it remains possible that Hippo signaling may modify YAP1 to affect the pathway studied herein, there is insufficient evidence to claim that Hippo signaling is involved. As such, the authors need to modify their assertion throughout the manuscript that RAS regulates Hippo signaling in this context.

There seems to be a slight misunderstanding here, which we suspect follows from a difference in how one defines 'the Hippo pathway'. We think of YAP as a target of the Hippo pathway. We provide evidence here that YAP is also a target of the Ras pathway, by a mechanism that is independent of the Hippo pathway. We did not claim that Ras regulates Hippo signaling, or that Hippo signaling is involved in Ras mediated transformation. Instead, our point was that by acting directly on YAP, Ras can override the tumor suppressive effects of the Hippo pathway, which are themselves mediated through regulation of YAP. Whether YAP is involved with other pathways is not really relevant here.

2. The authors extrapolate from engineered human cell lines and 293T cells to conclude that the observations presented herein are relevant to human tumors. This may well be the case but at a minimum, the authors should demonstrate that these relationships occur in tumors that have mutant RAS.

The relevance of our observations to RAS-driven tumor models is now well supported by the two recent Cell papers, in addition to our own work. Elevated YAP activity likely provides an advantage for tumors. Indeed, as shown here, and in the other papers, YAP can functionally replace RAS. Thus, we can expect selection in tumors for any number of mechanisms that can lead to increased YAP activity – low SOCS6 mRNA and/or protein levels among them.

We previously reported that depleting SOCS5 protein potentiated both mutant Ras-driven and endogenous EGFR-driven transformation of primary cells (Herranz & Hong et al., 2012). We observed low SOCS6 levels in colorectal tumors, in which KRAS is frequently mutant and in Glioblastoma, where KRAS is seldom mutated, but where the EGFR pathway is frequently activated. On this basis, we do not assume that the mutational status of RAS should be a critical factor. Indeed, we provide new data showing that the correlation between SOCS6 and the YAP target AREG seems to mainly come from the colorectal tumors that are wild-type for K-Ras and that there is no correlation in those that are K-Ras mutant (new fig 5D). Yet, the mechanism does function in some cancer cell lines that are K-Ras mutant (Fig 6F,G)

Moreover, the authors use different cell lines for the transformation experiments than are used for biochemical experiments (ubiquitination, protein-protein interaction studies) and most of the biochemical studies involve overexpressed proteins. To provide strong evidence that these pathways are truly operating in RAS transformed cells, the authors should use some of the same cell lines throughout the manuscript.

The cellular transformation assays require primary cells. Ras- or YAP-transformed primary cells were used for most of the shRNA experiments and SOCS overexpression experiments. We have added new data to figure 3(B) showing that YAP targets were upregulated in primary cells following SOCS6 depletion. This complements the luciferase reporter assay done in HEK293T cells. Primary cells are not well suited to ubiquitylation assays, which require efficient transgene expression in order to reach the necessary sensitivity. The Ubiquitylation mechanism was tested in HEK293T cells, similar to the Guan lab's work on YAP ubiquitylation by the Hippo pathway (Zhao et al 2010 G&D)

3. The authors assume that AREG is the key YAP1 regulated gene involved in this feedback loop. Based on what is presented, it is unclear how the authors came to this conclusion. How many other genes show a reciprocal relationship with SOCS6 levels? Was AREG the only one?

As was mentioned in the manuscript, we focused on AREG because of its known role as a mediator of YAP activity in regulation of the EGFR pathway (Zhang et al

2009). As seen in Figure 3B and 6A, AREG is among the more strongly upregulated YAP targets in primary cells transformed by Ras, or depletion of SOCS6. A number of other YAP targets show inverse correlations with SOCS6 in CRC tumors, but many do not. We have not investigated the functional significance of those YAP targets, and prefer not to go into them in this manuscript. The Hahn and dePinho lab papers highlight other YAP effectors with different biological functions. In light of our findings, linking Ras to regulation of YAP, the idea of a Ras/YAP/AREG/EGFR feedback loop seems to be of interest.

Furthermore, the authors have not demonstrated that YAP1 regulates AREG in this context nor have they shown that YAP1 and TEAD are present at the AREG gene. Although the data is consistent with this interpretation, it is impossible to know if this relationship is truly relevant and connected to changes in AREG levels.

AREG was identified as a YAP target by the Haber lab, working mainly in MCF10A cells (Zhang et al 2009). Figure 6A provides evidence that YAP overexpression induces AREG in primary BJ cells (this was 7A in the original ms). We now also show that YAP depletion reduces AREG levels in our primary cells (Fig 6B, new). We do not think it is important to repeat the Haber-lab's demonstration that YAP binds to the AREG promoter in primary cells: whether the regulation of AREG is direct or indirect does not affect our conclusions.

4. Related to point #3, the authors focus on glioblastoma and colon cancers, where they argue that EGFR signaling is important for the pathogenesis of these cancers. However, this manuscript is focused on RAS. Is there a correlation between SOCS6 levels and AREG in tumors that have mutant RAS? Is there a relationship between mutant KRAS and AREG?

We have examined SOCS6 and AREG levels with respect to KRAS status in 2 colorectal cancer datasets and added new data to Figure 5.

- Fig 5A shows that SOCS6 levels are significantly lower in tumors vs controls and that AREG levels are significantly higher in tumors vs controls.
- Fig 5B shows no difference in SOCS6 levels comparing K-Ras mutant tumors with wild-type K-Ras tumors (both datasets). AREG showed no difference in the TCGA dataset, but was higher in the tumors with wild-type K-Ras in the Gaedcke dataset (TCGA is more diverse; Gaedcke is rectal carcinoma only).
- Fig 5C shows an inverse correlation in the tumors between SOCS6 and AREG, when K-Ras mutational status was not considered.
- Fig 5D examines K-Ras status and the correlation between SOCS6 and AREG. The inverse correlation between SOCS6 and AREG was found only in the tumors with wild-type K-Ras ($p=0.0079$; $n=116$ tumors), but not in the K-Ras mutant tumors ($p=0.459$; $n=80$).

Even though there is no difference between SOCS6 and AREG levels comparing K-Ras mutant with wild-type K-Ras in the TCGA dataset, there is a significant difference in the correlation between SOCS6 and AREG levels in these tumors as a function of K-Ras status. This suggests an interesting hypothesis: the

SOCS6/YAP/AREG feedback mechanism may be important in tumors with normal K-Ras pathway activity, but less so in tumors adapted to chronic high-level activity associated with the K-Ras mutant.

5. The authors never specify which RAS allele they are using. Based on citations, one might assume that they have used HRAS but this is never stated. Are the same findings true if one uses KRAS or NRAS?

H-Ras G12V was used (apologies for the oversight, I thought we had mentioned this). We also provide data showing that codon-optimized G12V mutant versions of N-Ras and K-Ras have comparable effects of cellular transformation assays, and on regulation of SOCS mRNA level. New data are in supplemental Figure E1.

6. The authors argue that a RAS-SOCS5/6-YAP1-AREG loop is critical for RAS induced transformation. How does RAS regulate SOCS5/6? Are the well-studied RAS effector pathways (MEK, PI3K, RAL) important for this regulation?

We have tested Ras^{V12} mutants specific for the different effector pathways (H-Ras G12V E37G, H-Ras G12V Y40C and H-Ras G12V T35S) on SOCS6 mRNA.

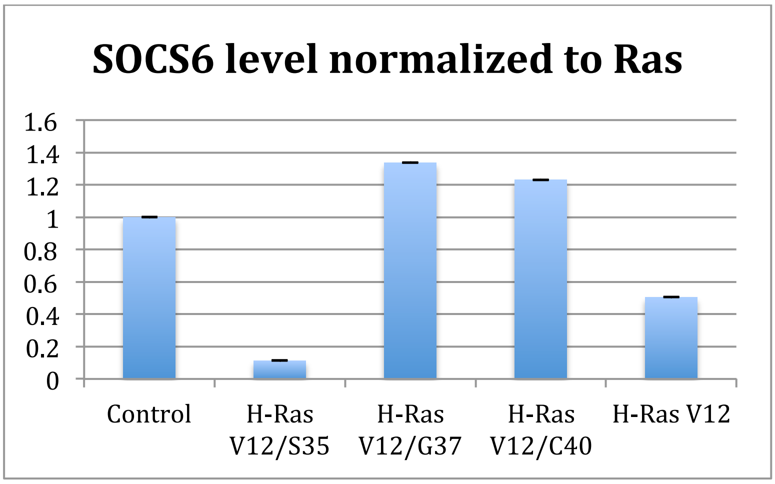

RNA was measured by QPCR in cells transduced to express the indicated proteins. SOCS6 mRNA was normalized to the level of Ras mRNA expression.

It appears that the T35S mutant form of Ras was most effective at lowering SOCS6 levels. T35S can act via the Raf effector pathway, but not via the Ral or PI3K pathways. We agree that it would be interesting to explore this in more depth, but not in this paper.

7. In general, the studies demonstrating ubiquitination of YAP1 by SOCS5/6 are incomplete. What form of ubiquitination is regulated by SOCS5/6? What sites in YAP1 are critical for this modification. Is phosphorylation necessary to allow ubiquitination? Without this information, the reader is left wondering how direct the effect of SOCS5/6 are on YAP1.

SOCS proteins have been identified as substrate recognition factors for ElonginBC/cullin5 ubiquitin ligase complexes. Based on this hypothesis, we showed:

- co-IP data linking HA-tagged SOCS6 and endogenous YAP (Fig 4A as well as

co-IP of the two tagged proteins).
- depletion of SOCS5/6 reduced YAP ubiquitylation (Fig 4B).
- overexpression of SOCS5/6 increased YAP ubiquitylation (Fig 4C).
- that these interactions do not depend on the phosphorylation sites that mediate interaction with the bTrCP/SCF ubiquitylation complex.
- that ElonginBC/cullin5 activity are involved in YAP ubiquitylation (Fig 4G).

All together these data make a fair case that SOCS6 has a role for as a substrate recognition factor for YAP to mediate its degradation by an ElonginBC/cullin5 ubiquitin ligase complex. We agree with the reviewer that it would be interesting to explore in more depth how SOCS recognizes YAP, and to examine what kind of ubiquitylation etc. Again, we think that is beyond the scope of this paper. Would knowing more about this really affect the key conclusions?

Minor points
1. The introduction is written quite bluntly and reads as a series of declarative statements.
Noted.

2. Figure 4C is of poor quality
Noted, but the result is not ambiguous. It is confirmed in Fig 4E (in which the blot is cleaner).

3. Figure 6B. The p value seems to overstate the relationship shown. Have the authors accounted for multiple hypothesis testing?
In the new Figure 5C, D, we use Spearman correlation analysis to assess the significance of the relationship between SOCS6 and AREG levels. The p-value doe not assess how tight the correlation is, but allows us to assess whether the relationship is statistically significant. The Spearman-r value reflects the scatter in the data. The correlations are not tight, which we take to mean that other factors affect the expression of these two transcripts. The correlations are statistically significant and we provide experimental evidence that they are functionally significant in the primary cell transformation assay.

4. The interaction of SOCS5 and SOCS6 is interesting but underdeveloped? Do these proteins form a complex that is required for ubiquitination?
It is an interesting question, but one that we think does not need to be addressed in this manuscript.

Ref 2

Hong et al have described a novel mechanism by which the oncogenic Ras promotes stabilization of the Hippo pathway mediator YAP1. They find that activated Ras opposes the Hippo-driven destruction of YAP1 by regulating the ubiquitin ligase complex substrate recognition factors SOCS5/6, which bind YAP1 to promote its ubuitination. The results are supported by a variety of overexpression and RNAi studies in cultured fibroblasts, and are extended to xenografts, and several human cancer cell lines.

Overall, the findings are of topical interest, especially in light of 2 recent related publications in Cell describing the role of YAP1 in bypassing a requirement for activated KRAS in maintaining malignant transformation. Here, the authors essentially build on that novel Ras-YAP1 link with an additional mechanistic aspect.

There is, however, a notable lack of somewhat standard controls throughout this manuscript. In particular, most of the shRNA and many of the overexpression/ectopic expression studies do not include protein blotting data to support the expected effects on expression of YAP1, Ras, TEAD, AREG, LATS2, and SOCS5/6. In a few cases, RNA levels are shown, but it's protein that really matters.

We have a somewhat different view than the reviewer on the relevance of RNA and protein controls for the RNAi experiments. RNAi leads to depletion of the mRNA. Hence the most direct control for efficacy of the treatment is to measure target mRNA level. The RNAi reagents are well-validated in our hands and in the literature for their ability to reduce expression of these proteins. Once a given shRNA has been shown to deplete the protein, RNA controls suffice.

RNAi controls:

YAP: YAP RNAi efficacy was shown in Fig 2B by qPCR. We provide the immunoblot for this figure in Figure E4 and a second example in Figure 6B showing the effect of YAP depletion on AREG levels.

LATS2: shRNA mediated depletion of the protein was shown in Fig 2C.

SOCS6: shRNA mediated depletion of the protein was shown in Fig 3A. The mRNA knockdown by Q-PCR was shown in Figure E1. For Fig 6A, RNA was isolated from a subset of the cells prepared for the colony formation assay shown in Fig 3A. The immunoblot control for SOCS6 depletion is shown in Fig 3A. We have added shRNA and siRNA depletion of SCOS6 protein in cancer cells in Fig E10.

AREG: AREG shRNAs are validated by immunoblot in Fig 6C.

Overexpression controls:

YAP blots are shown for Figs 2D, 4B, C, D, E & G (Flag-YAP in D,E,G). Fig 6C is similar to Fig 2D. We do not have the YAP blot for Fig 6C. The blot for a similar experiment is provided in Fig E11.

Ras: We have done experiments with Ras over-expression many times. Ras expression is required for colony formation (example shown for the reviewer). For Fig2A, we did not show a Ras overexpression blot, but we did show the effect of Ras expression on ERK pathway activity. In our view this should suffice.

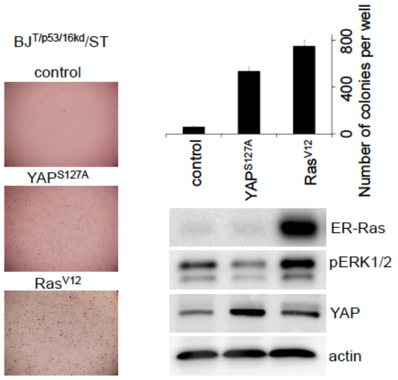

TEAD-DN overexpression was used as a positive control for the experiment in Fig 2B in which YAP shRNAs were used to deplete YAP and block colony formation. I'm sorry, but we do not have a TEAD blot for this control. We didn't think it was necessary since it is a well-established reagent in the literature (e.g. Liu-Chittenden et al 2012 Genes Dev 26: 1300-1305). The reagent performed as expected in the control. If the lack of the TEAD immunoblot is a problem, we can remove the positive control from the figure. We prefer to leave it in, but will follow the editor's wishes on this.

LATS2: overexpression was only used as a positive control for the luciferase assay in Fig 3E. The ability to LATS to work in this assay is well documented in the literature. We don't have the LATS immunoblot for this experiment. Since the point it made was a non-essential confirmation of what is shown in Fig 3D, we have removed LATS2 from the figure. If the editor requests, we can put it back.

S**OCS6** Immunoblots showing SOCS6 expression were provided for many experiments.

- Figure 1C: the SOCS6 blot shown in Fig 3D was from the cells in this experiment. We now mention this in the figure legend.
- Figures 4D, 5A and E5: immunoblots were already provided.
- Figures 4C/4E: We now provide the SOCS6 blot for 4E, but we do not have a SOCS6 blot for Fig 4C. 4C was done before we had functional antibody to SOCS6. The result is confirmed in Fig 4E which shows that SOCS6 overexpression increases ubiquitylation of YAP.
- Figure 4F: we do not have the blots for this panel. However, given that we see elevated colony formation, we can infer that YAP was expressed, and similar to Fig 1C, expression of SOCS6 blunted this effect, so we can infer that SOCS6 was expressed in this assay as well.

Which isoform of Ras is being used here? Probably H-Ras, since there is transforming activity in fibroblasts (which Kras largely lacks). Of course, Kras is the more relevant human oncogene.

H-Ras G12V was used (apologies for the oversight, I thought we had mentioned this). It is true that K-Ras normally transforms poorly. It is poorly translated, due

to rare codon usage. Using partially codon-optimized versions of N and K Ras (Curr Biol. 2013 23:70-5), we find that G12V mutant versions of N-Ras and K-Ras have comparable effects of cellular transformation assays, on regulation of SOCS mRNA level and on YAP protein. New data are in supplemental Figure E1.

The link to EGFR is weak, and the claim in the abstract that Rasv12 "activates the endogenous EGFR pathway" should be removed. The authors' inference that this pathway is involved (last paragraph of Results) is not supported by anything other than effects on ampiregulin expression, and the AREG shRNA study (which lacks protein expression controls). In fact, this is readily testable with EGFR kinase inhibitors. And, in my view, this is unlikely to work, especially when considering that EGFR inhibitory drugs have not proven to be clinically effective in Ras mutant cancers.

AREG has been shown previously to act via activation of the endogenous EGFR pathway, downstream of YAP (Zhang et al 2009). In that paper, the Haber lab showed that EGFR inhibitors blocked the proliferation inducing activity of YAP in MCF10 cells, including growth in 3D culture (similar to the soft agar colony formation assay). Figure 6B (new) shows that depletion of YAP by shRNA treatment reduces AREG levels in our primary BJ cells (including protein expression controls). Figure 6C (new) shows that depletion of AREG by shRNA treatment reduces activation of the EGFR receptor in primary BJ cells. Figure E11 (new) shows that treatment with antibodies to EGFR blunts soft agar colony formation by cells expressing YAP. All this provides further support for our interpretation of the previous data.
