# Supplementary material for: Opposing activities of the Ras and Hippo pathways converge on regulation of YAP protein turnover
Source: EMBO J. 2014 Sep 1;33(21):2447–57. doi: 10.15252/embj.201489385 (PMC4283404; doi:10.15252/embj.201489385)

Fig (2A)

First probed with anti-YAP (rabbit), then membrane is stripped and probed with anti-tubulin (mouse)

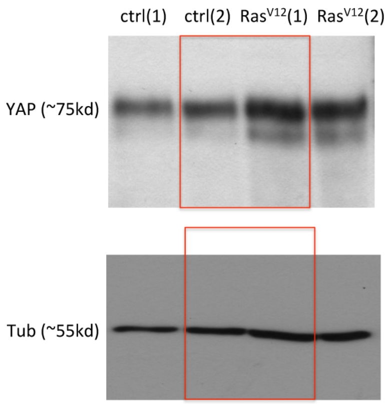

Fig (2A)

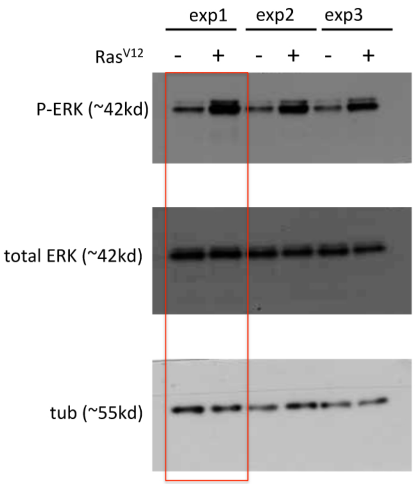

First probed with anti-pERK (mouse), then membrane is stripped and probed with anti-total ERK (rabbit). The membrane is stripped fro another time and probed with anti-tubulin (mouse)

Fig (2A)

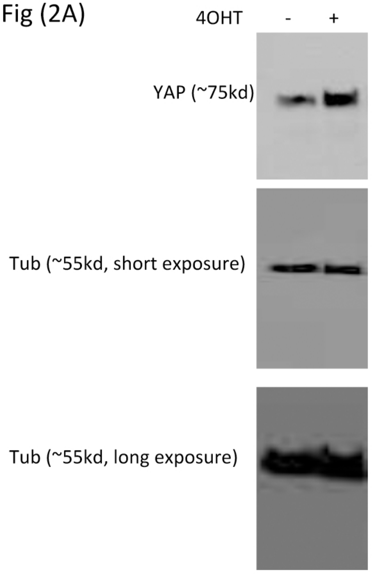

The membrane is first probed with anti-YAP (rabbit), then membrane is stripped and probed with anti-tubulin (mouse). Both long and short exposure of tubulin are shown

Fig 2C

membrane cut into two, upper one is used to probe for lats2; lower one is firstly used to probe p-YAP (rabbit) and tub; then stripped to probe with total YAP (mouse)

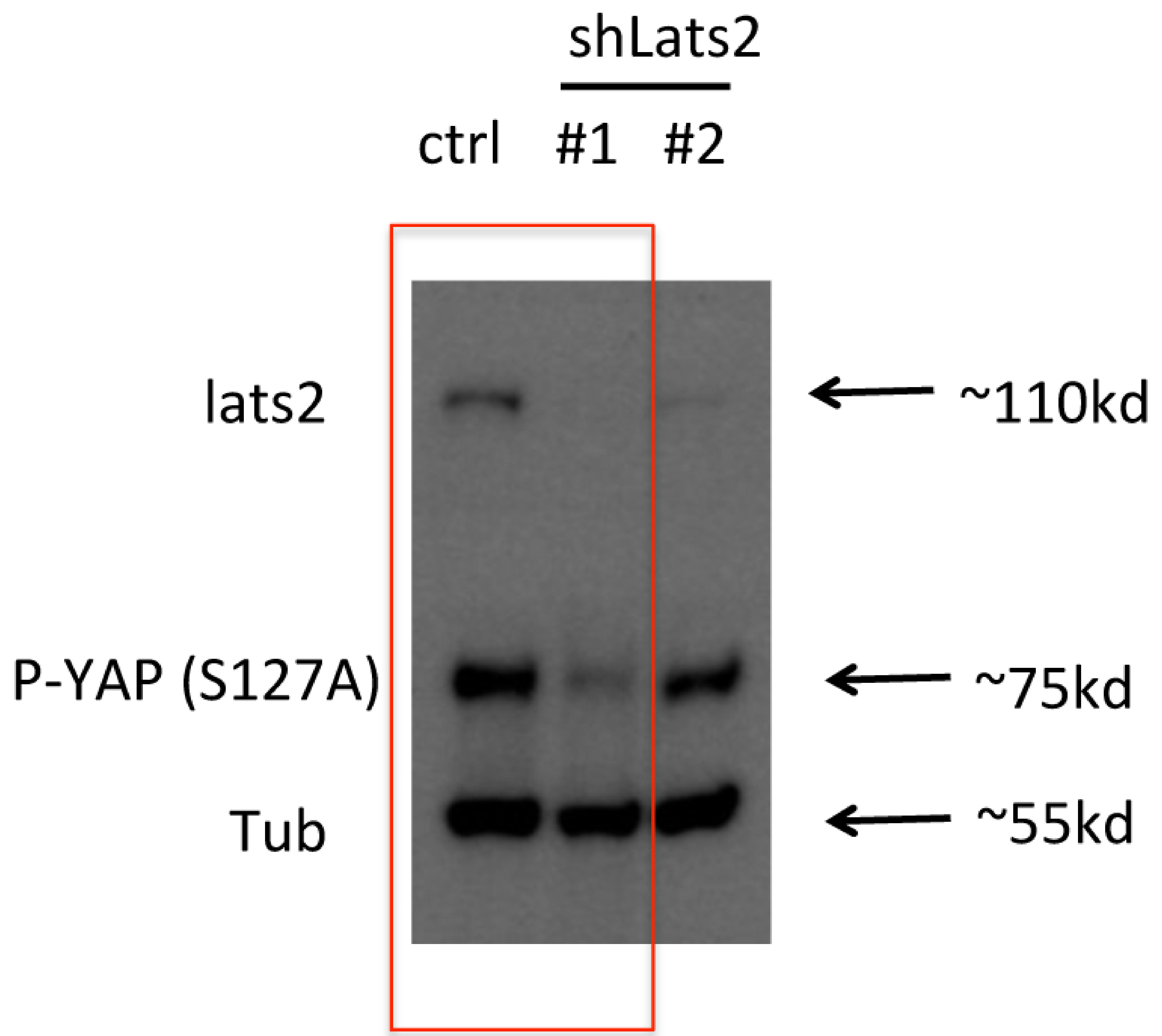

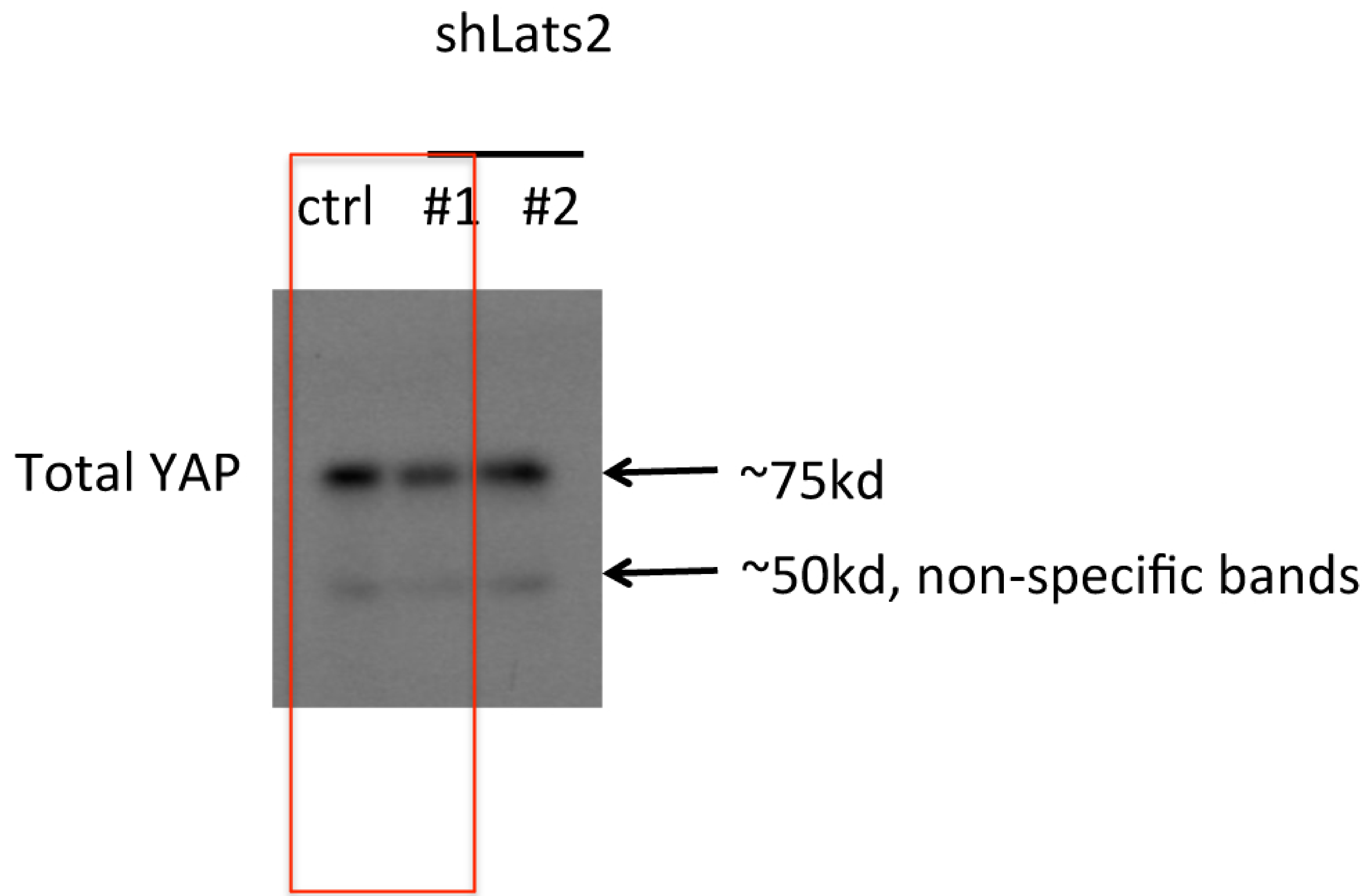

Fig 2D The membrane is probed with rabbit anti-FLAG and mouse anti-tubulin together

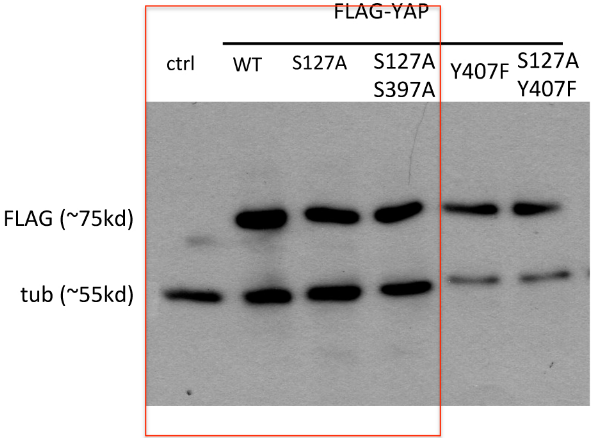

Supplement: Supplementary file 2 [file embj0033-2447-sd2.pdf]
