# Supplementary material for: Opposing activities of the Ras and Hippo pathways converge on regulation of YAP protein turnover
Source: EMBO J. 2014 Sep 1;33(21):2447–57. doi: 10.15252/embj.201489385 (PMC4283404; doi:10.15252/embj.201489385)
Supplement: Supplementary file 3 [file embj0033-2447-sd3.pdf]

Fig 3A short exposure

Antibody used: rabbit anti-YAP
Mouse anti-tubulin

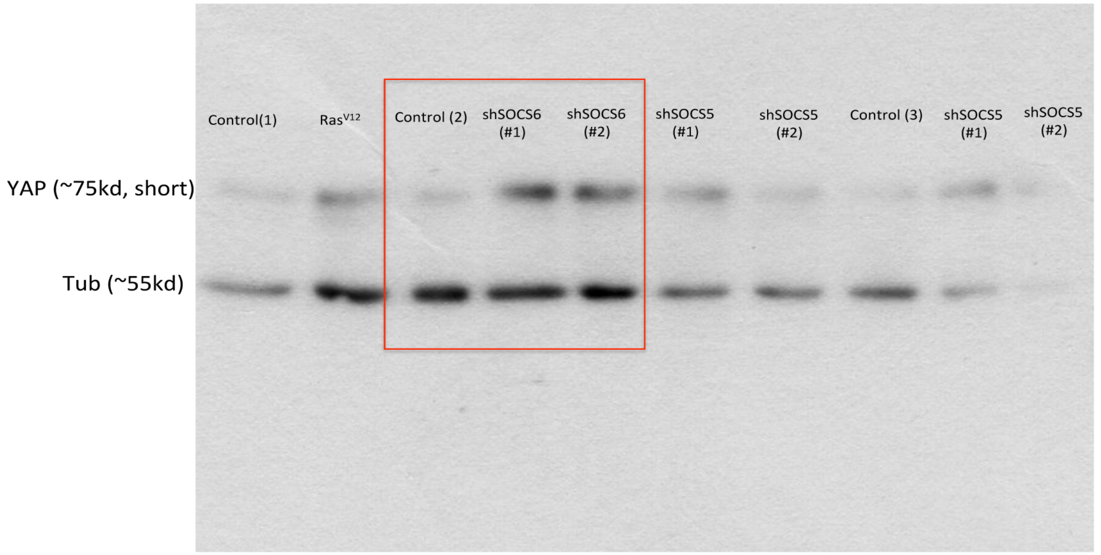

Fig 3A long exposure

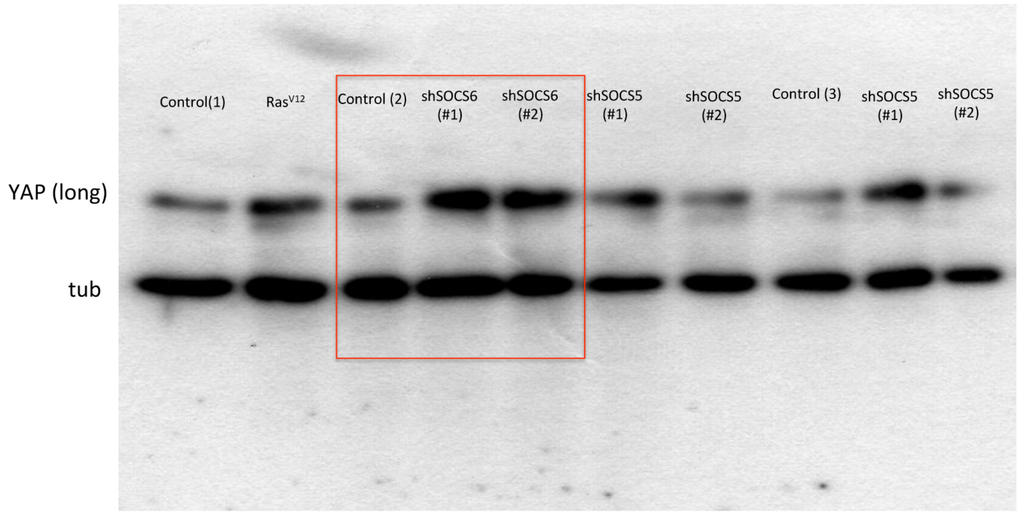

Fig 3A

Membrane was probed with rabbit anti-SOCS6
and mouse anti-tubulin together

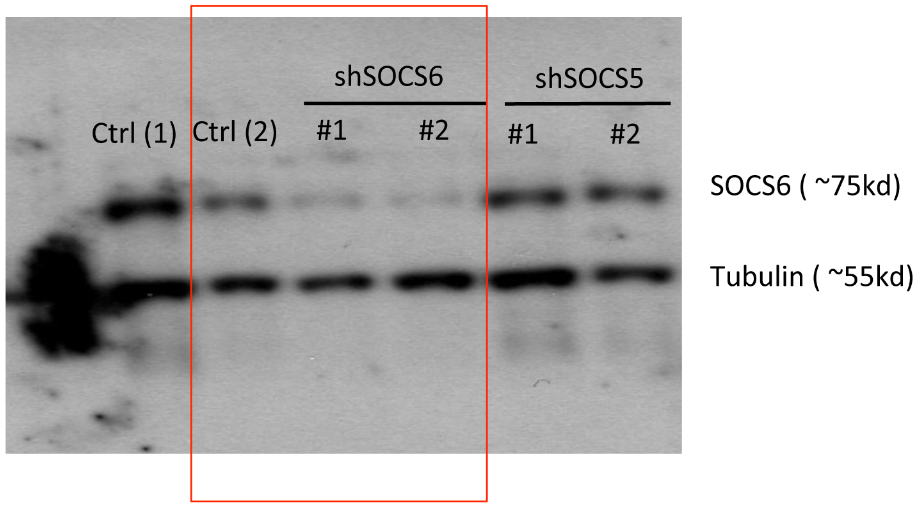

Fig 3E

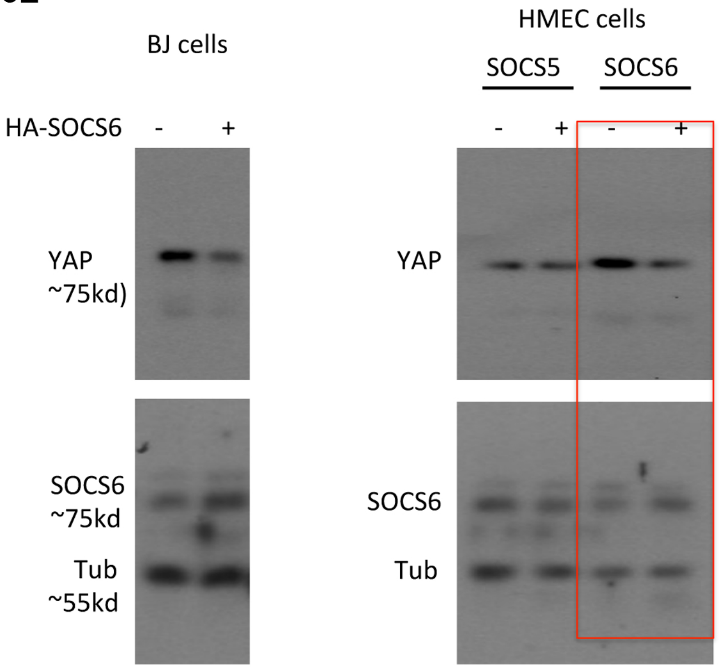
BJ cells
HA-SOCS6
-
+
YAP
~75kd)
SOCS6
~75kd
Tub
~55kd
HMEC cells
SOCS5
SOCS6
-
+
-
+
YAP
SOCS6
Tub
