# Supplementary material for: Opposing activities of the Ras and Hippo pathways converge on regulation of YAP protein turnover
Source: EMBO J. 2014 Sep 1;33(21):2447–57. doi: 10.15252/embj.201489385 (PMC4283404; doi:10.15252/embj.201489385)

Fig 4A

YAP IP (rabbit anti-YAP)

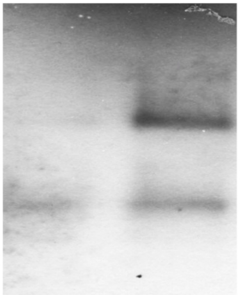

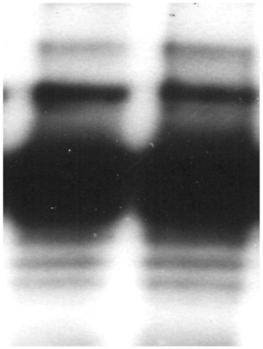

Fig 4A Input (total lysates)

Antibody used:

1. Rabbit anti-YAP
2. Mouse anti-tubulin
3. Mouse anti-HA

Membrane first probed for YAP and tubulin;
Then membrane was then stripped and probed for HA

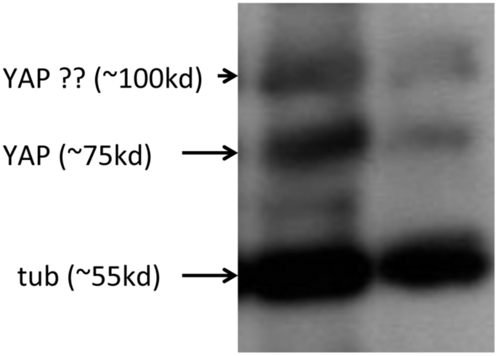

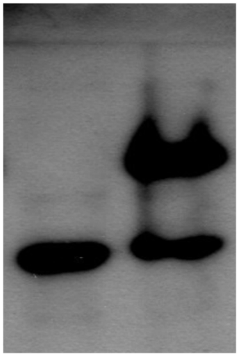

Fig 4D

FLAG IP (rabbit anti-FLAG), membrane probed with anti-HA (rabbit)

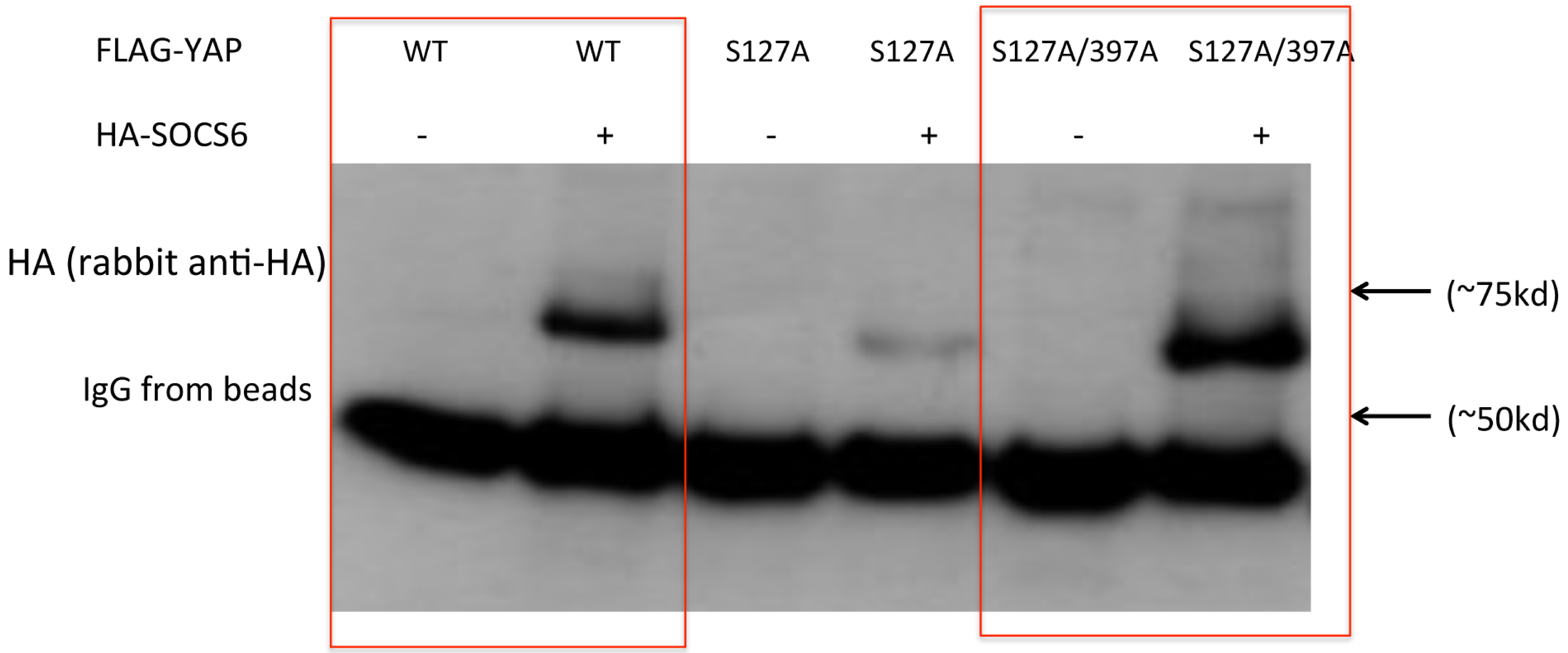

FLAG IP : Membrane probed with anti-FLAG (rabbit)

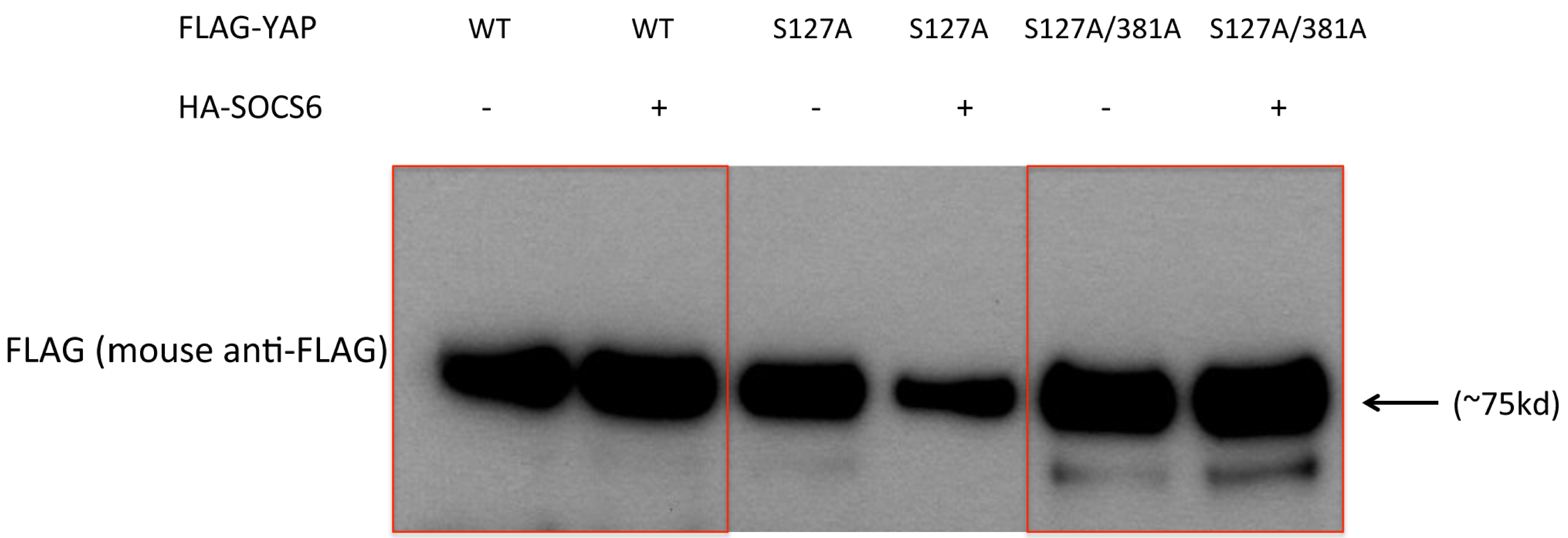

Fig 4D

Inputs (independent membranes)

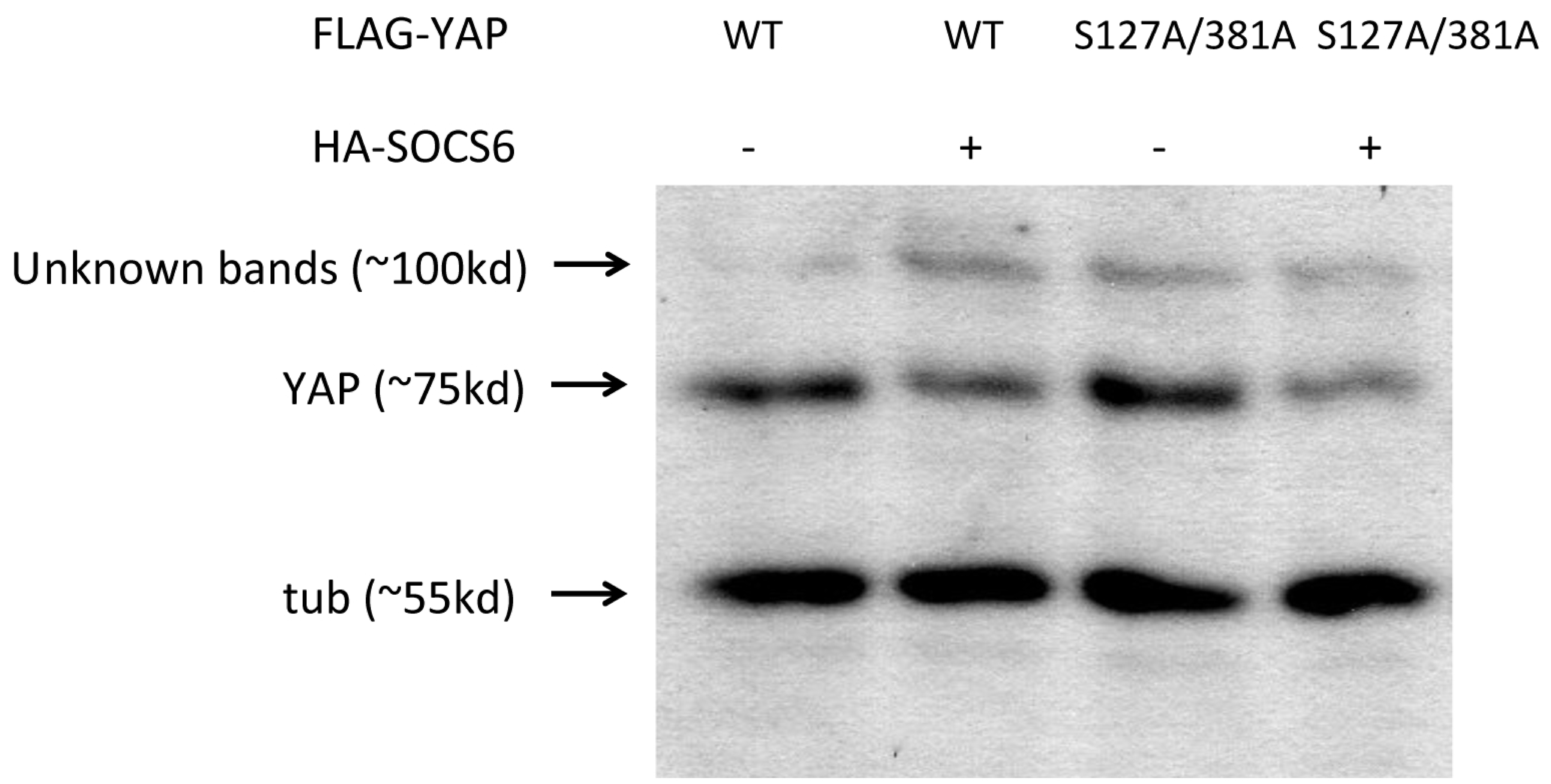

Fig 4E

Antibody used :

1. Rabbit anti-c-myc tag
2. Rabbit anti-SOCS6

First probed for c-myc-ubi

then stripped and probed with FLAG

After another stripping , probed for SOCS6 (20 min exposure)

IgG →
From beads

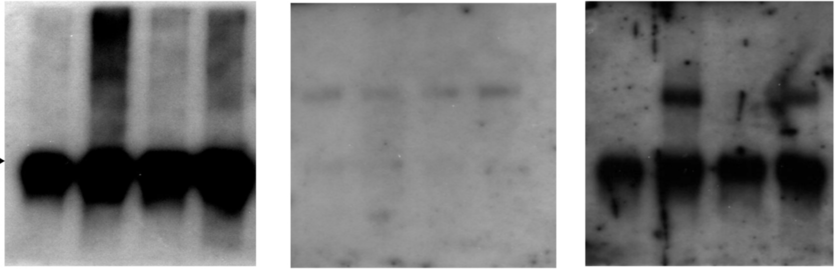

Fig 4G

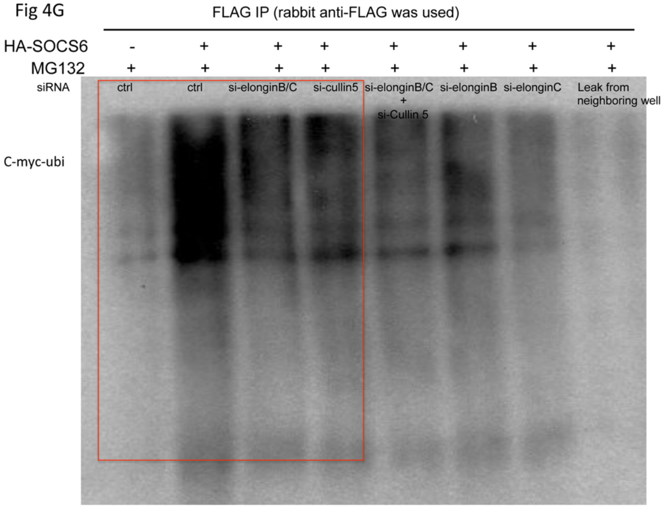

Fig 4G

Antibody used after stripping : mouse anti-FLAG, in this exp, beads were removed from supernatant by centrifugation

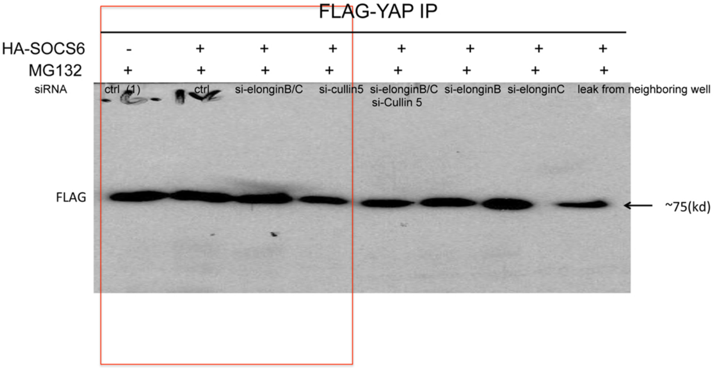

Fig 4G

Antibody used after stripping: rabbit anti-SOCS6, in this exp, beads were removed from supernatant by centrifugation

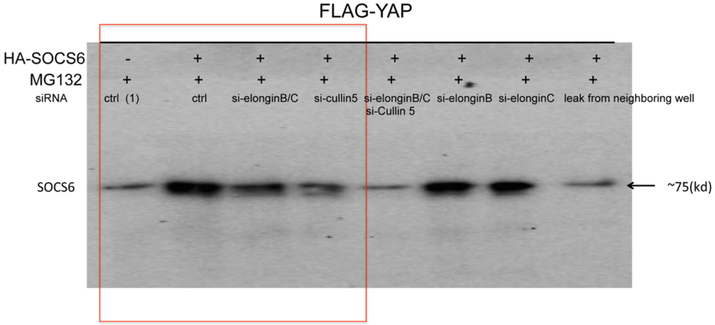

Supplement: Supplementary file 4 [file embj0033-2447-sd4.pdf]
