# Supplementary material for: Opposing activities of the Ras and Hippo pathways converge on regulation of YAP protein turnover
Source: EMBO J. 2014 Sep 1;33(21):2447–57. doi: 10.15252/embj.201489385 (PMC4283404; doi:10.15252/embj.201489385)
Supplement: Supplementary file 5 [file embj0033-2447-sd5.pdf]

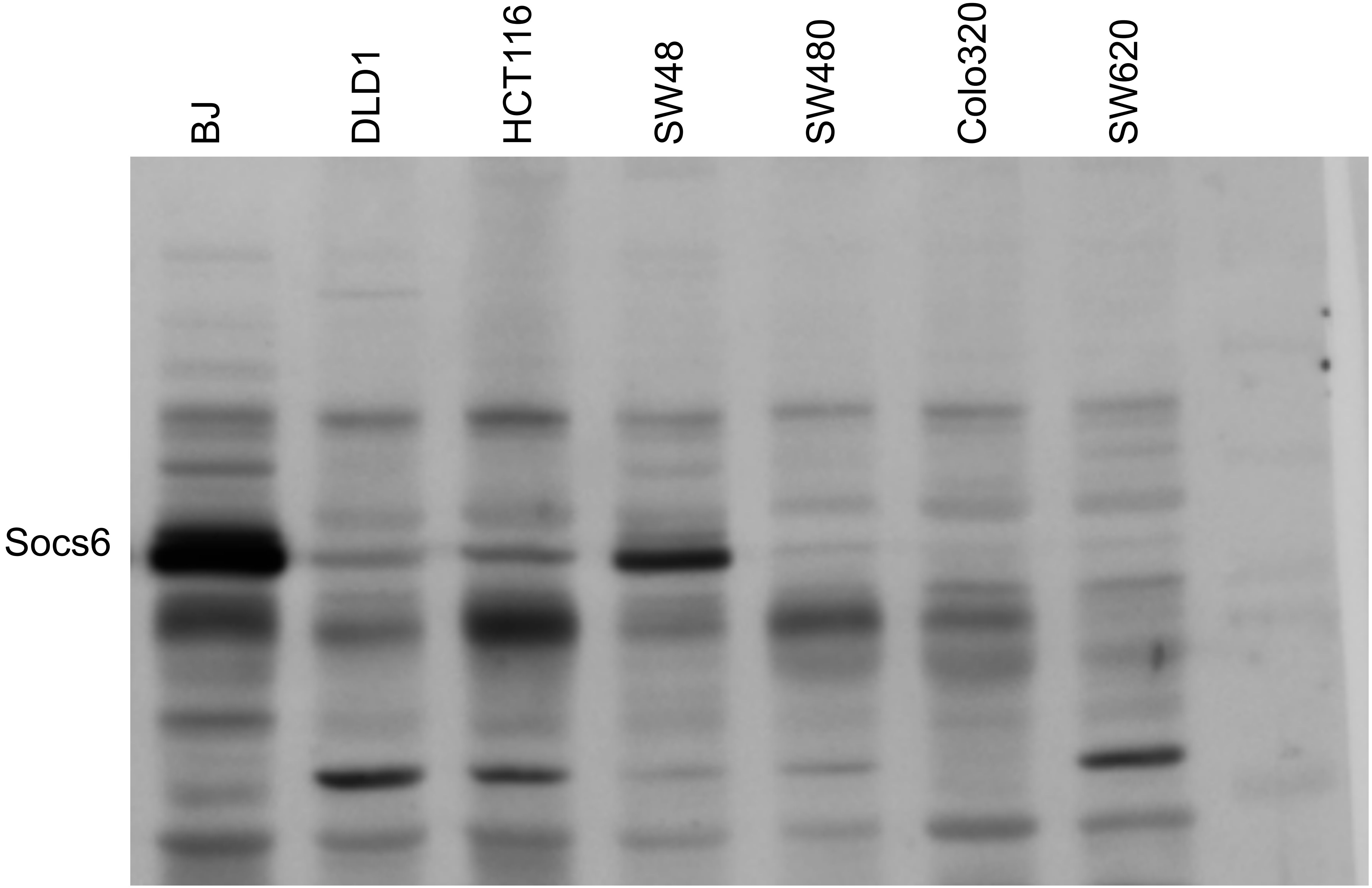
BJ
DLD1
HCT116
SW48
SW480
Colo320
SW620
Socs6

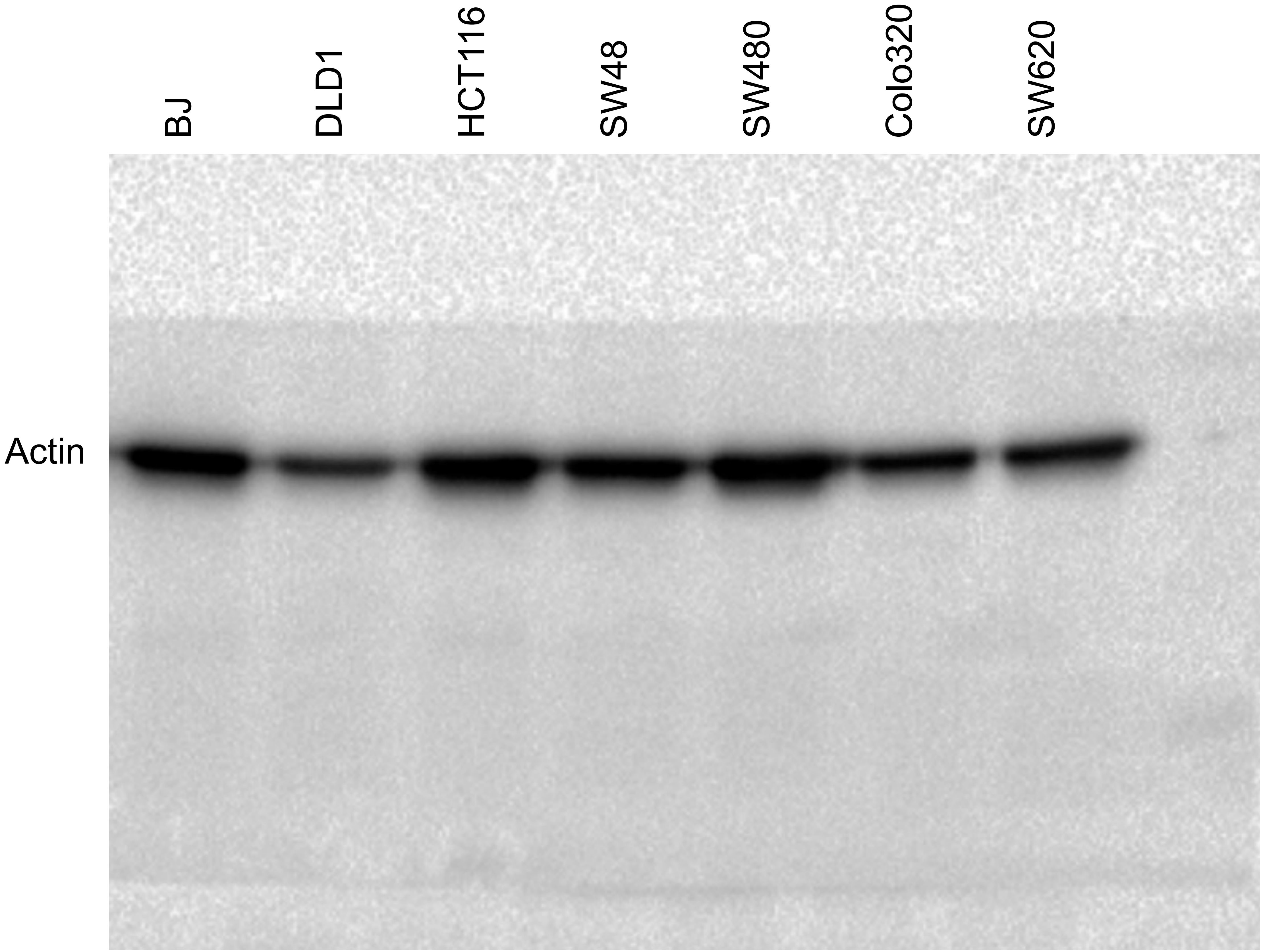
BJ
DLD1
HCT116
SW48
SW480
Colo320
SW620
Actin

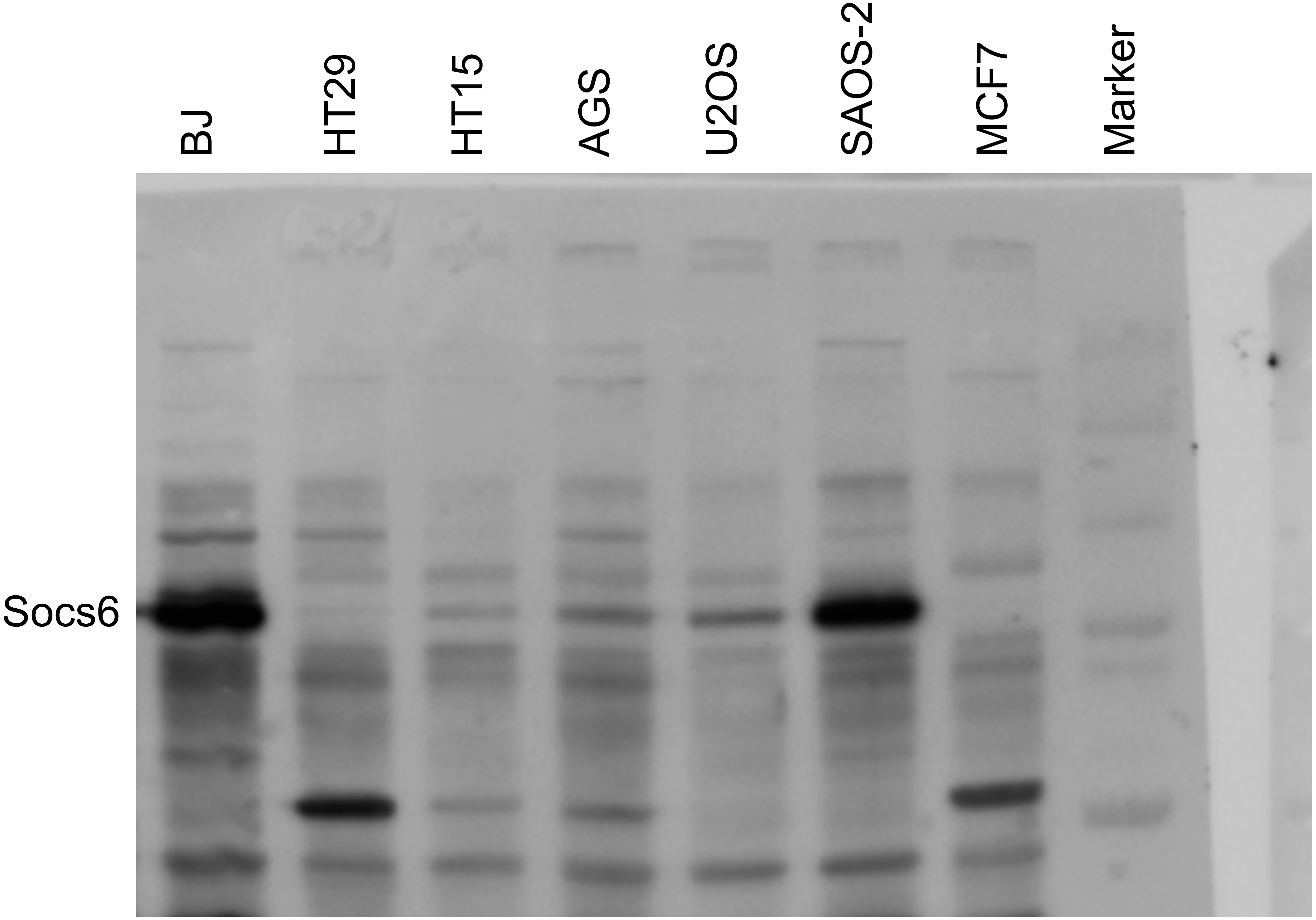

BJ
HT29
HT15
AGS
U2OS
SAOS-2
MCF7
Marker
Socs6

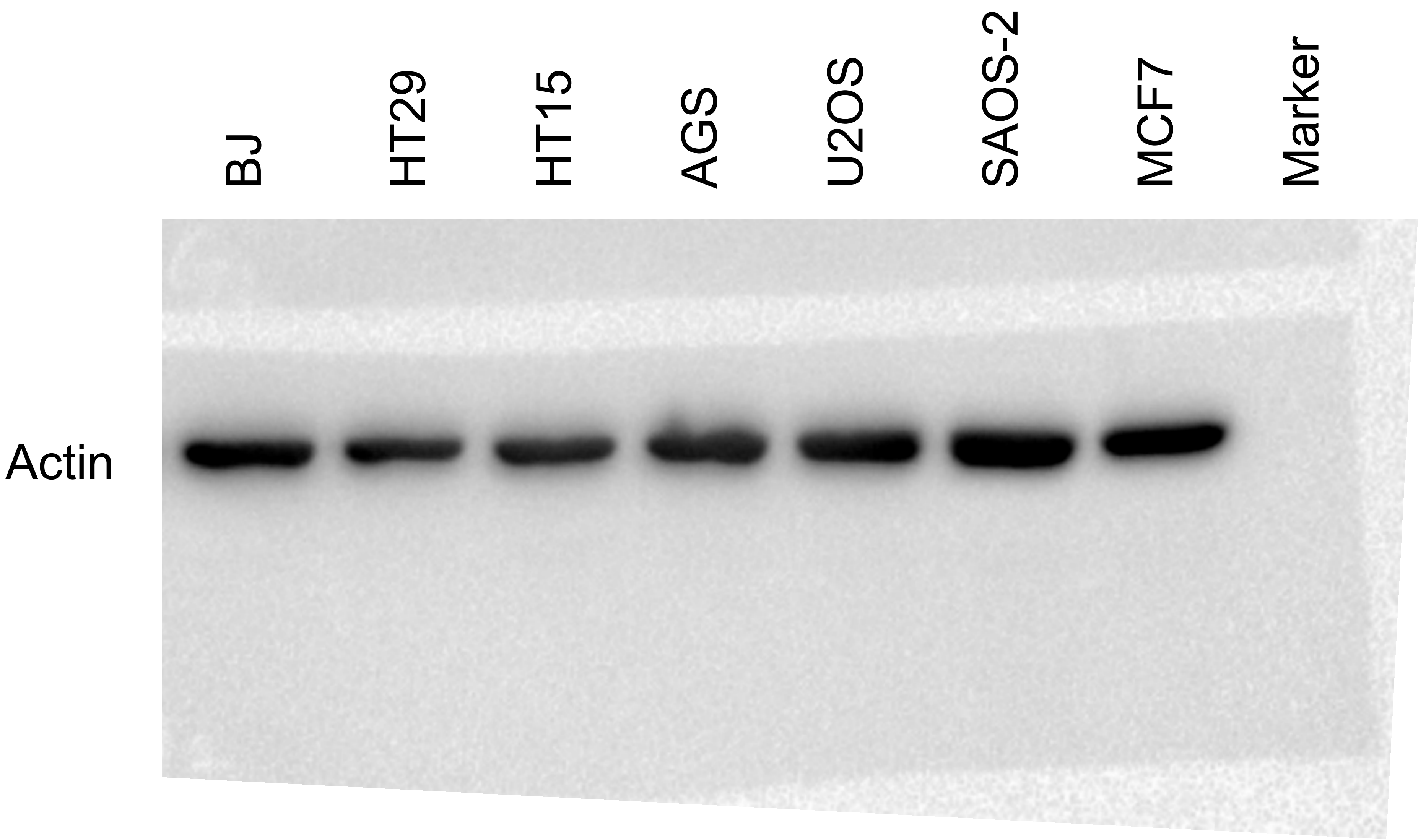
BJ
HT29
HT15
AGS
U2OS
SAOS-2
MCF7
Marker
Actin

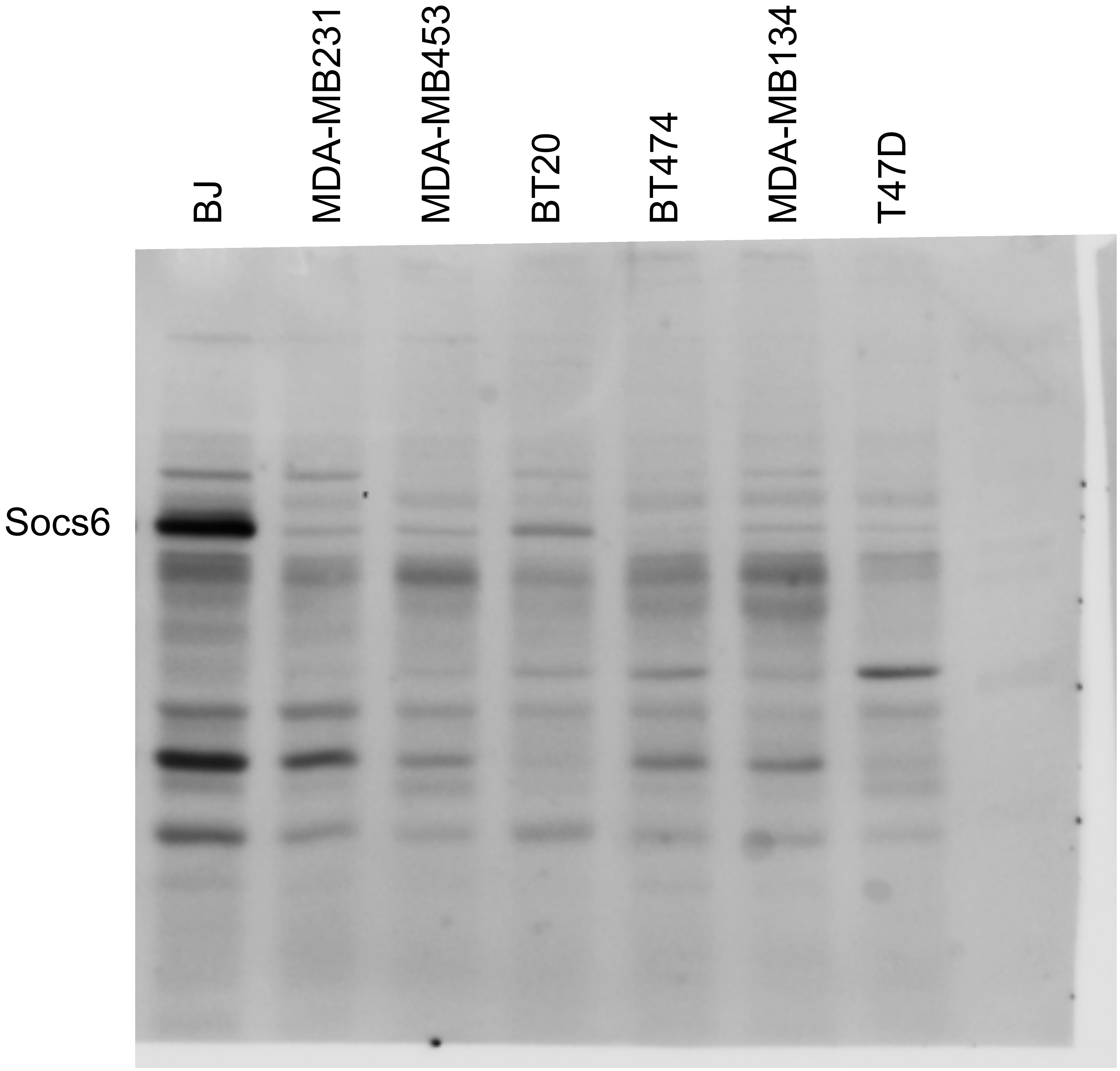
BJ
MDA-MB231
MDA-MB453
BT20
BT474
MDA-MB134
T47D
Socs6

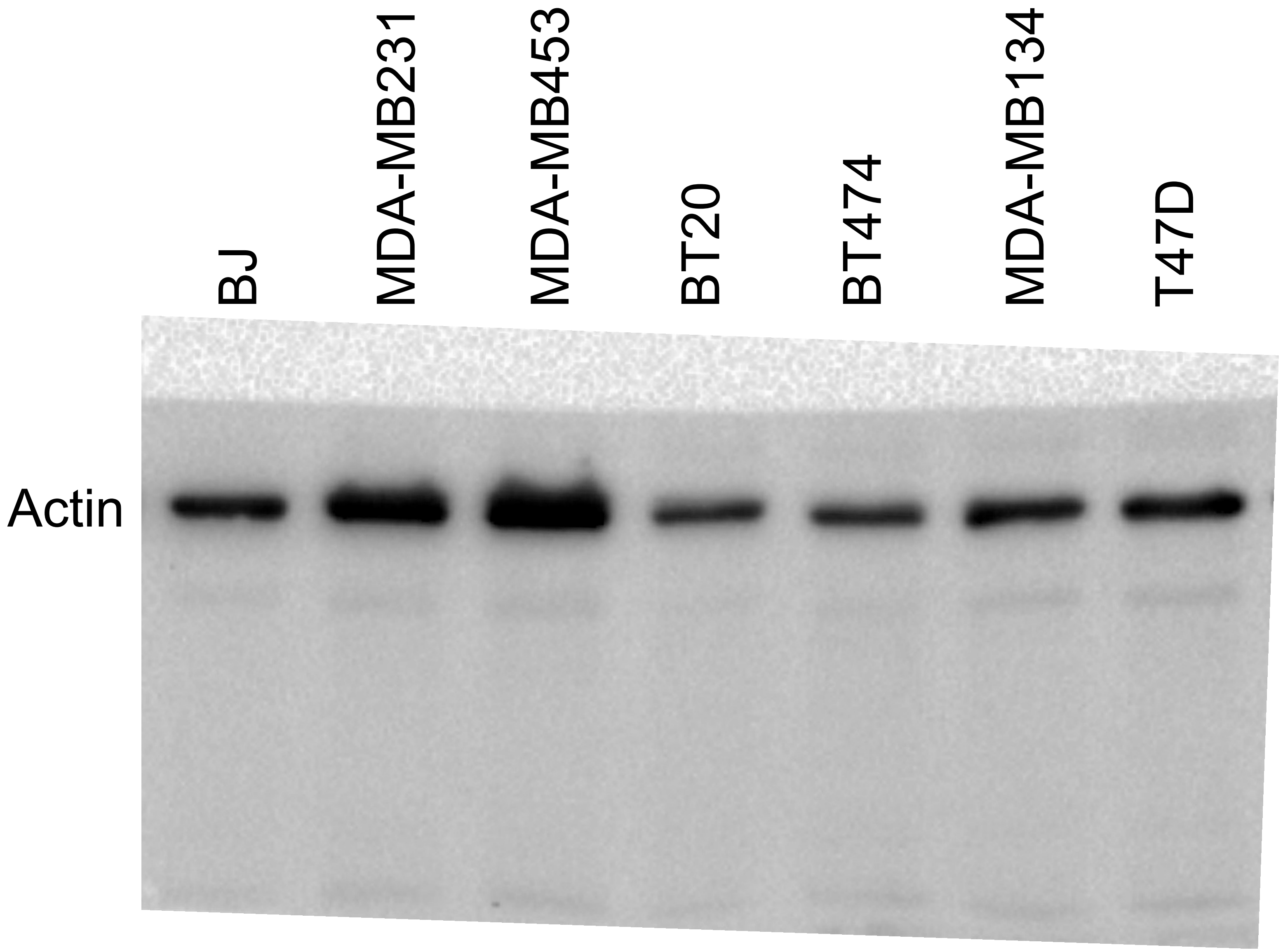
BJ
MDA-MB231
MDA-MB453
BT20
BT474
MDA-MB134
T47D
Actin

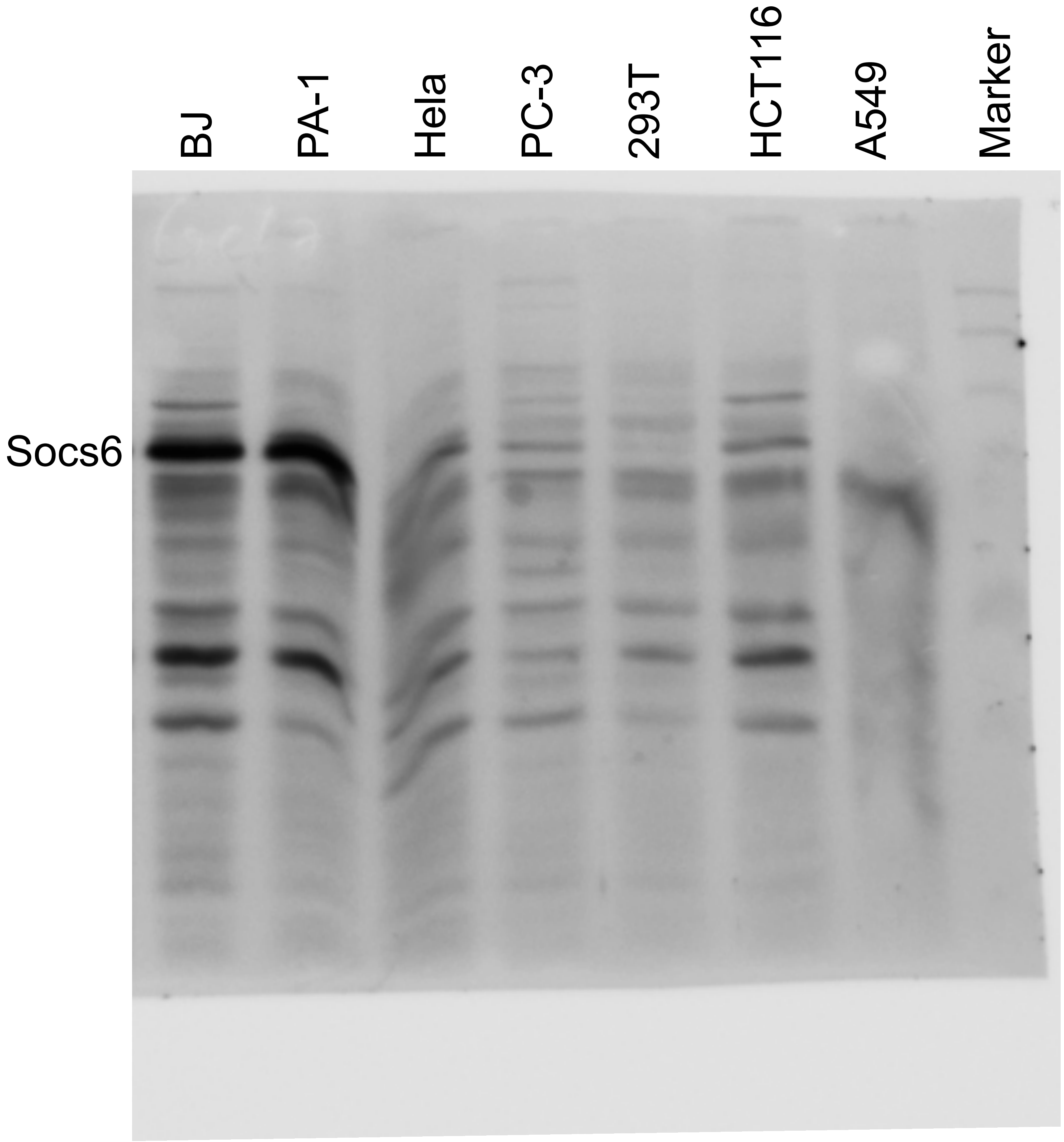
BJ
PA-1
Hela
PC-3
293T
HCT116
A549
Marker
Socs6

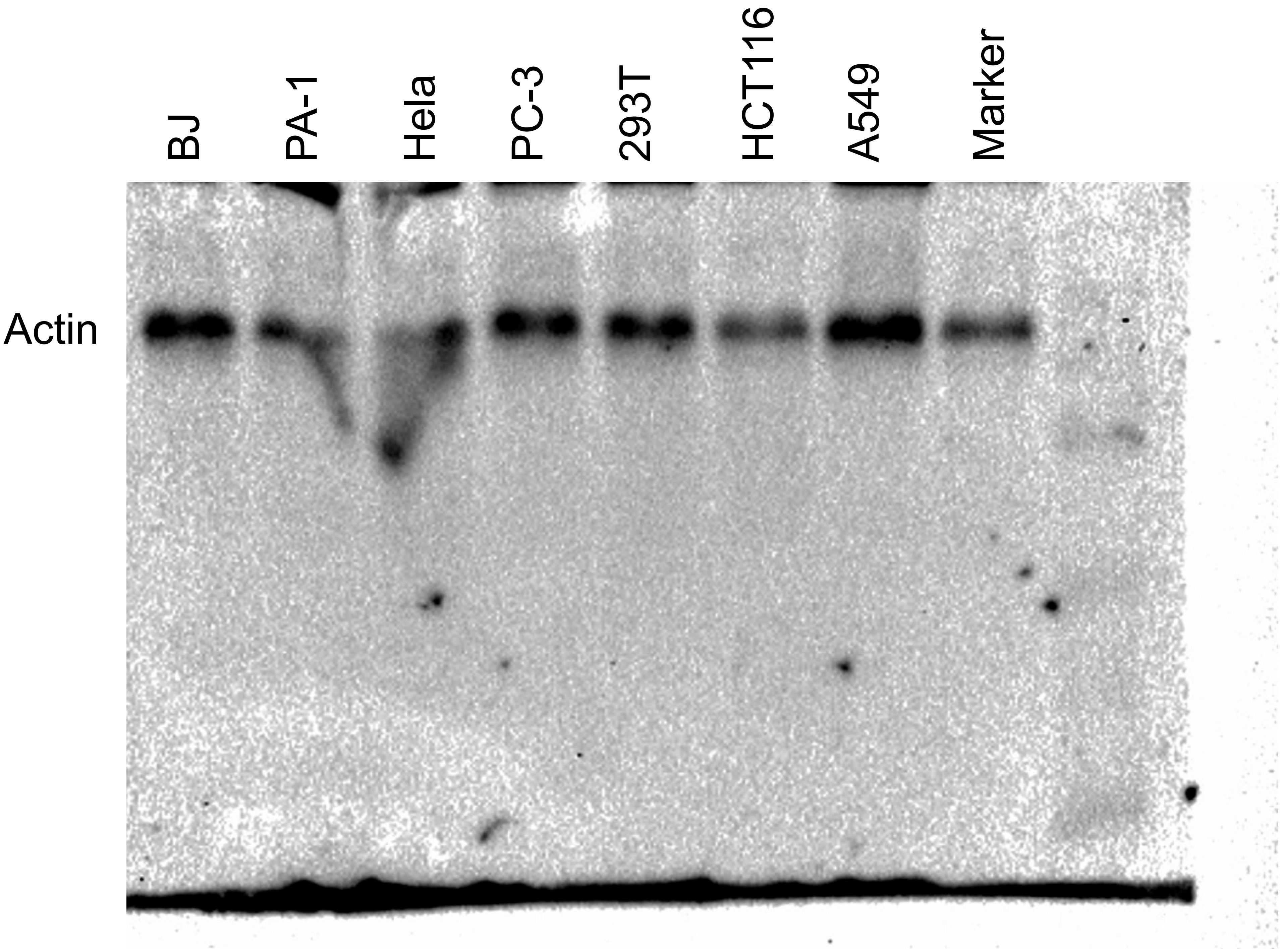

BJ
PA-1
Hela
PC-3
293T
HCT116
A549
Marker
Actin
