# Supplementary material for: Opposing activities of the Ras and Hippo pathways converge on regulation of YAP protein turnover
Source: EMBO J. 2014 Sep 1;33(21):2447–57. doi: 10.15252/embj.201489385 (PMC4283404; doi:10.15252/embj.201489385)
Supplement: Supplementary file 6 [file embj0033-2447-sd6.pdf]

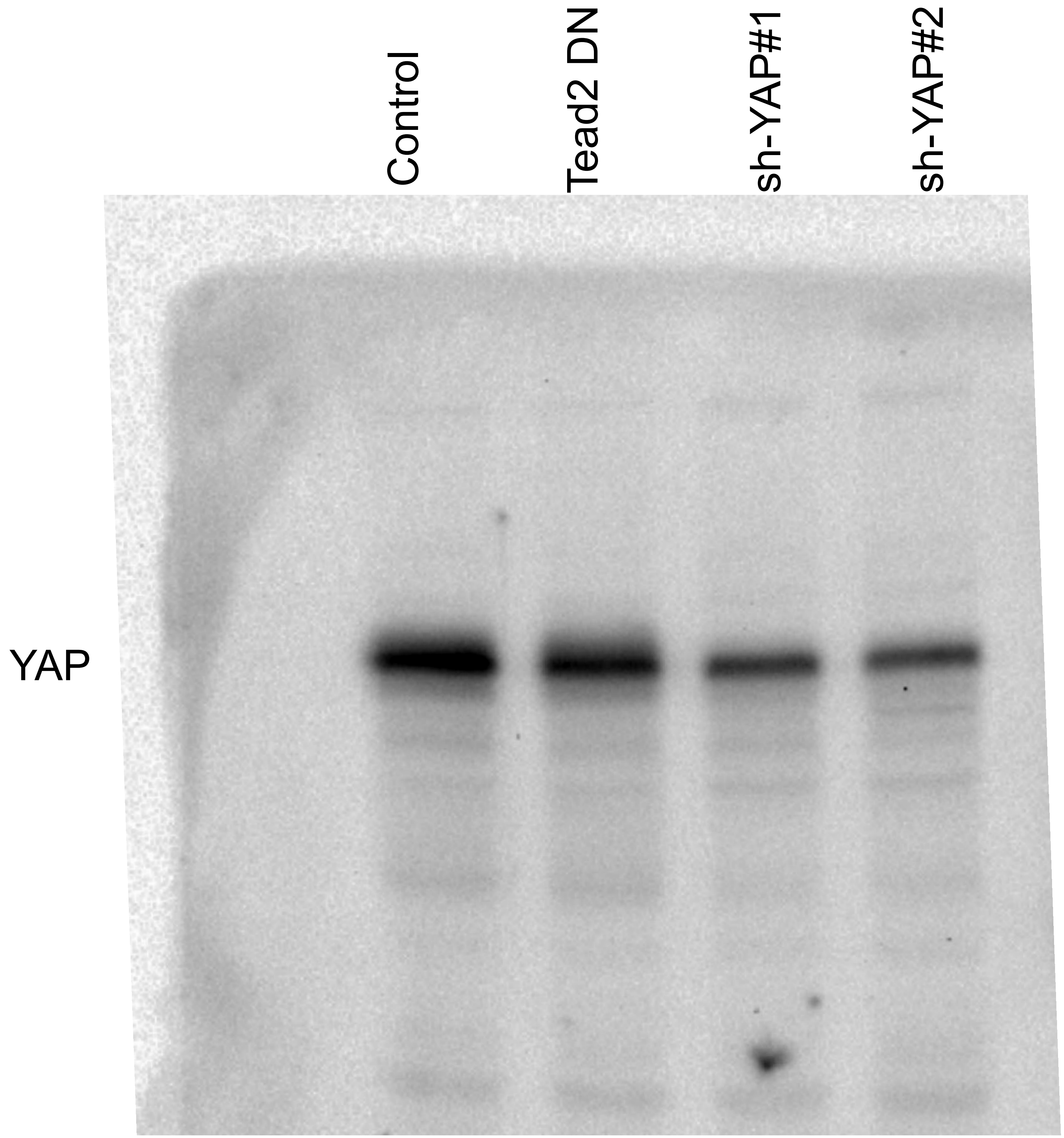

Cells: $BJ^{T/p53kd/p16kd/ST}$

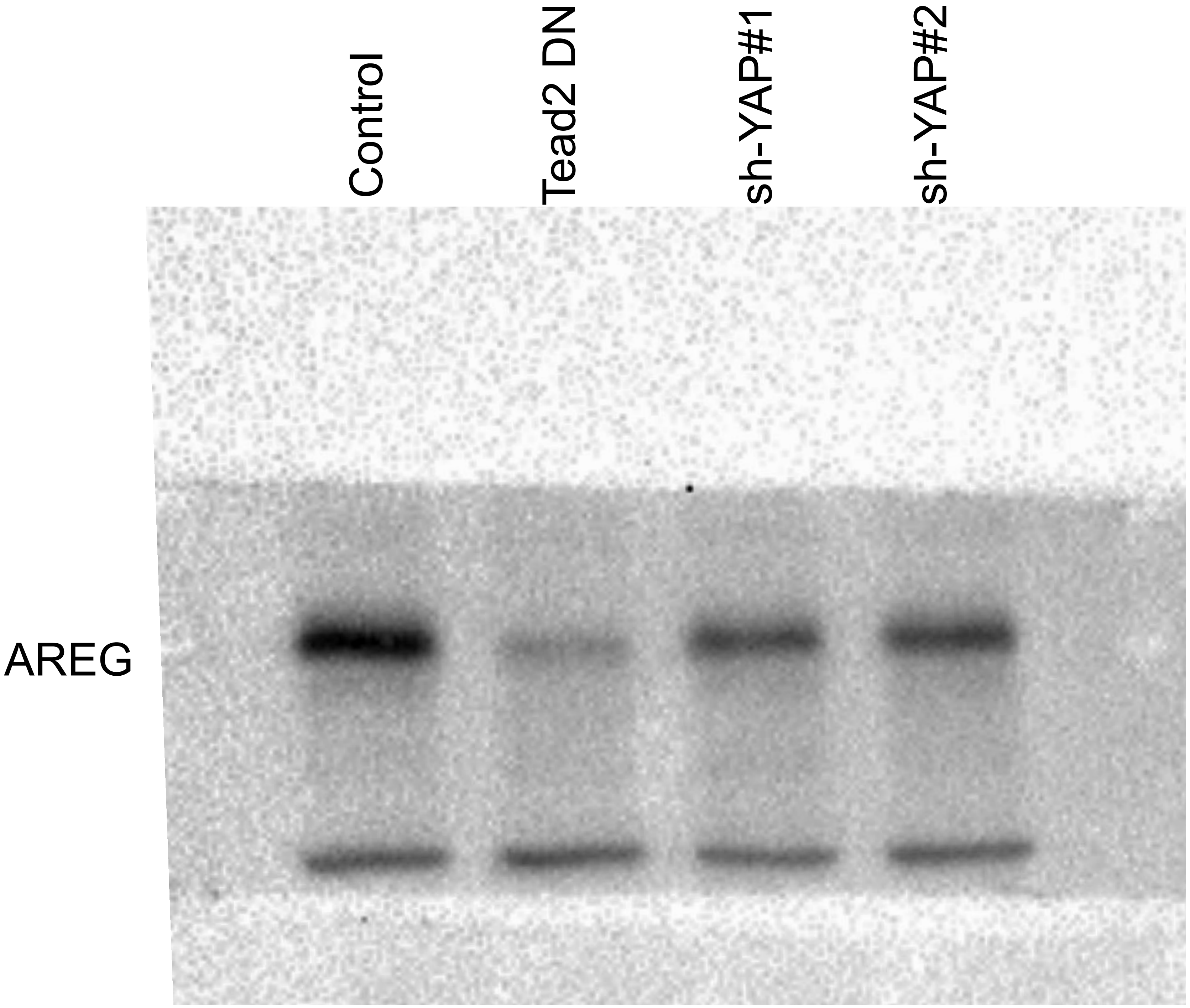

Cells: BJ$^{T/p53kd/p16kd/ST}$

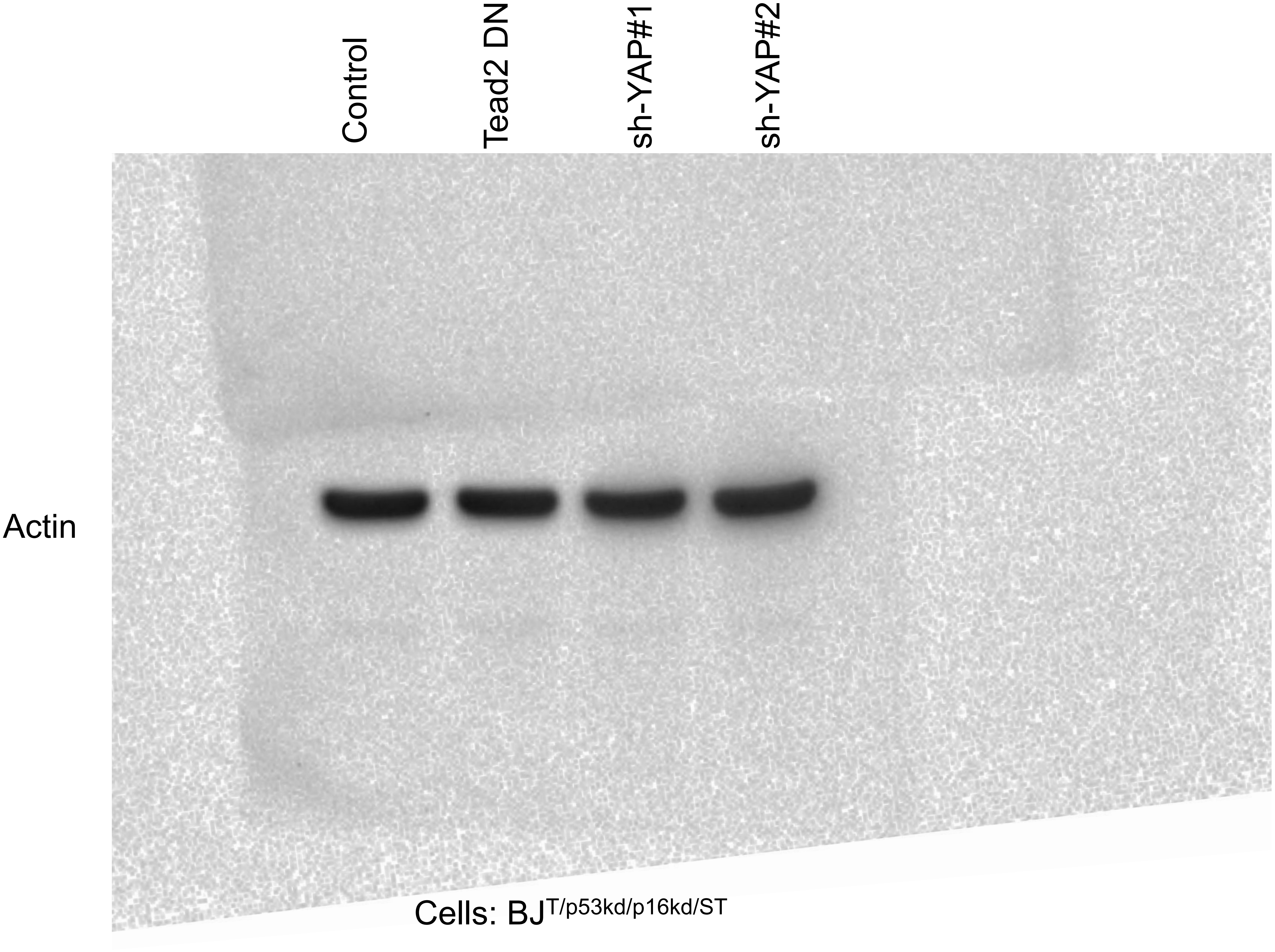
Control
Tead2 DN
sh-YAP#1
sh-YAP#2
Actin
Cells: BJ$^{T/p53kd/p16kd/ST}$

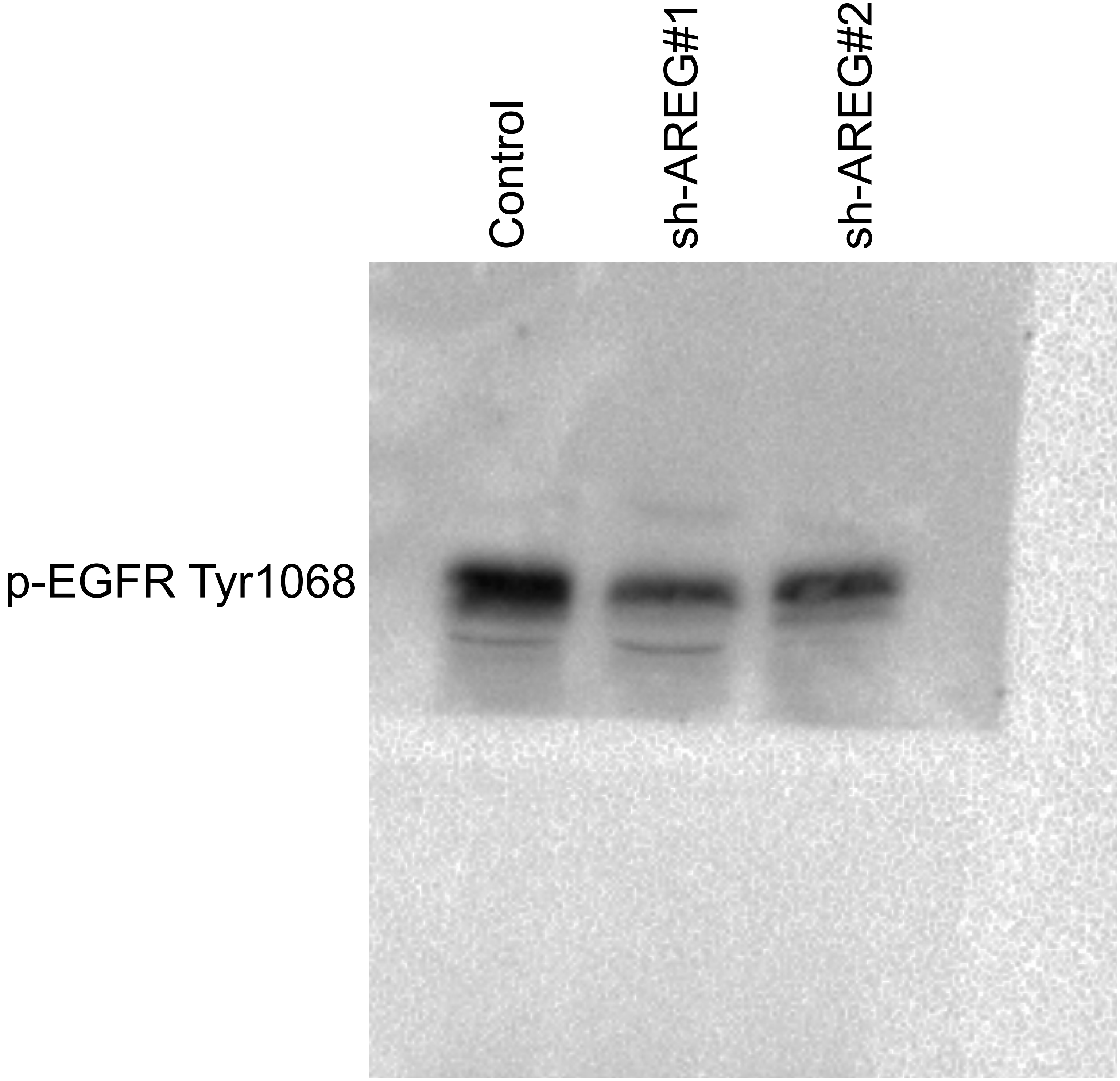

Cells: $BJ^{T/p53kd/p16kd/RasV12/ST}$ 6% gel

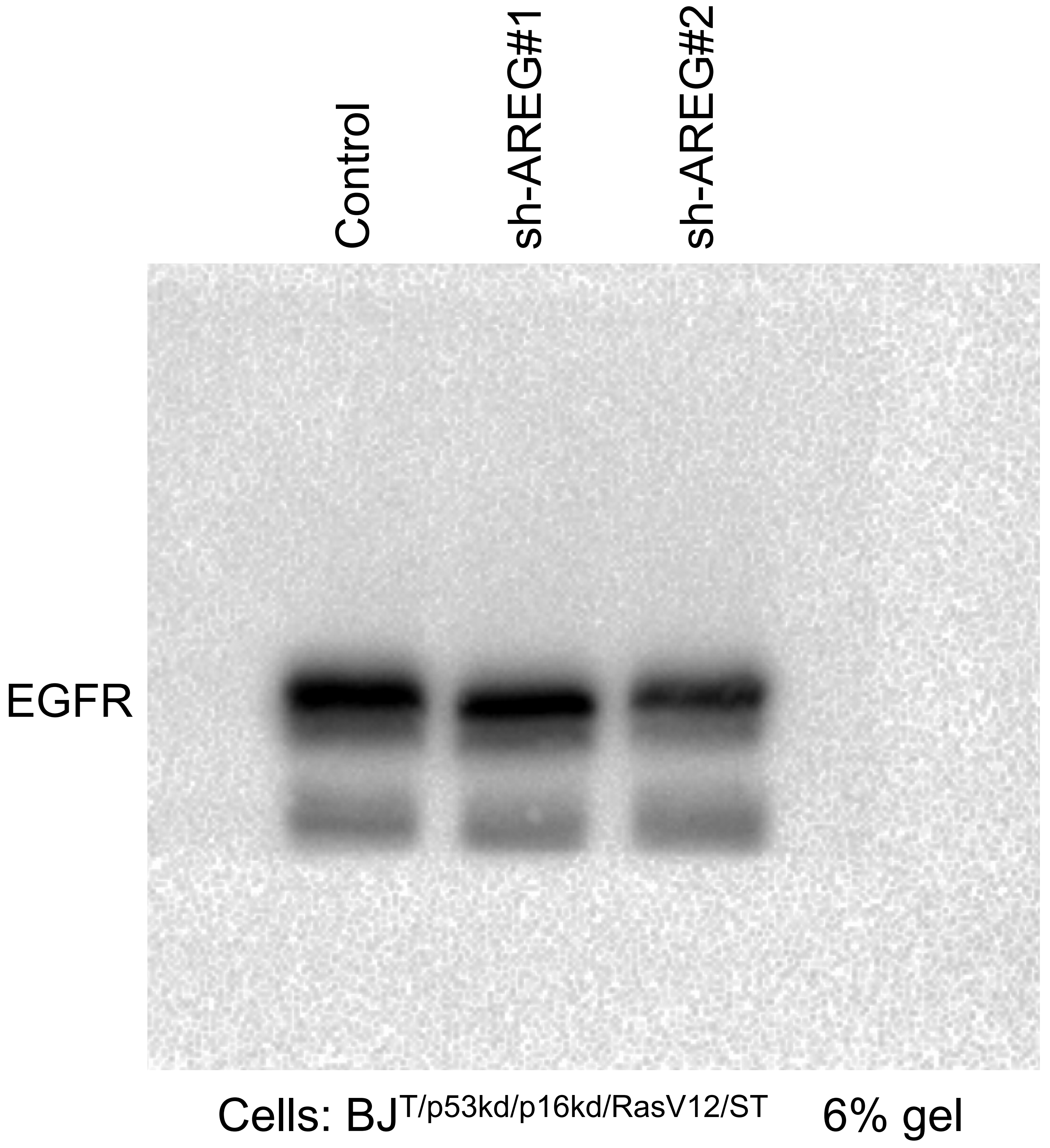
Control
sh-AREG#1
sh-AREG#2
EGFR
Cells: BJ^T/p53kd/p16kd/RasV12/ST
6% gel

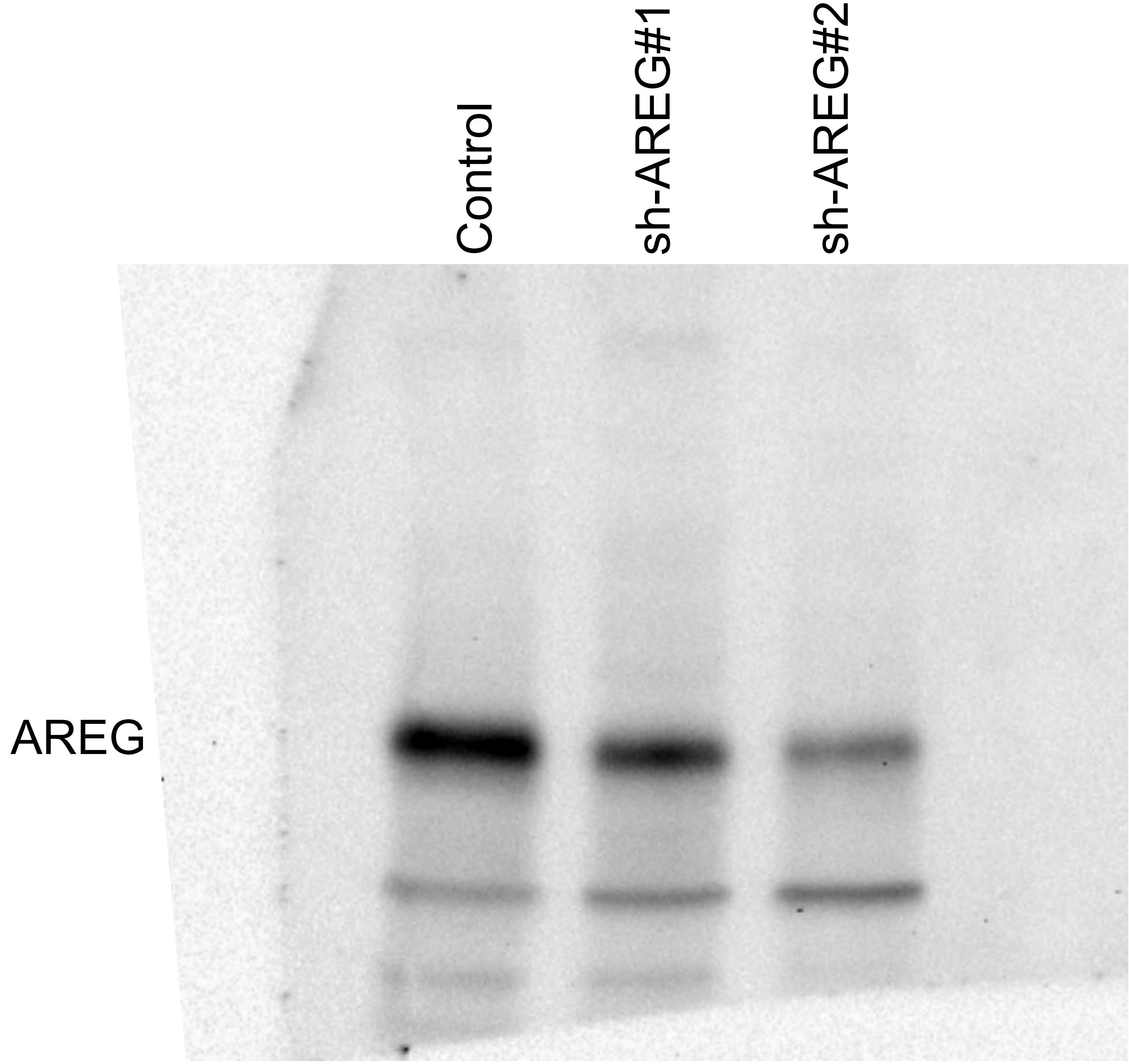

Cells: BJ$^{T/p53kd/p16kd/RasV12/ST}$ 14% gel

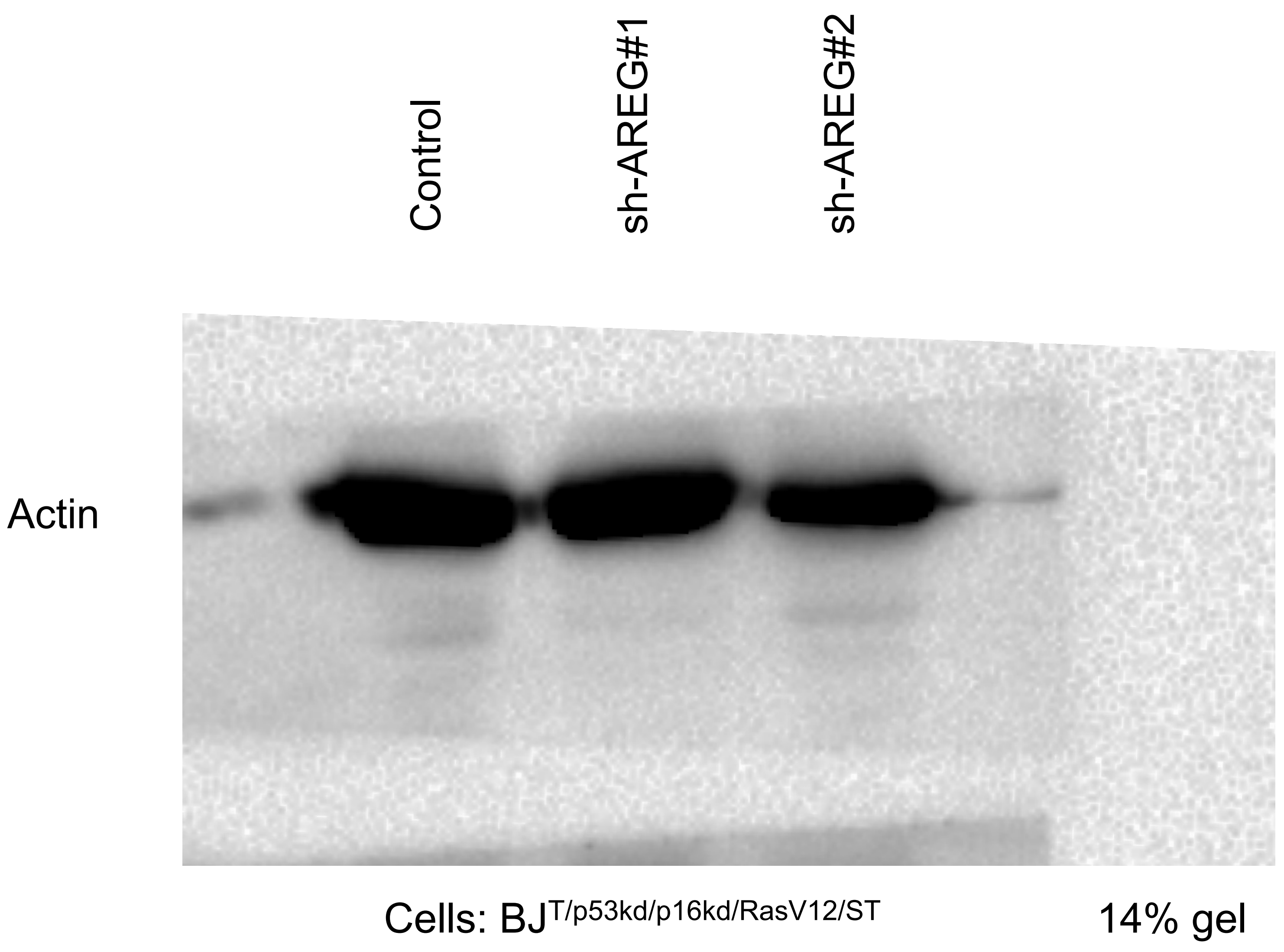
Control
sh-AREG#1
sh-AREG#2
Actin
Cells: BJ T/p53kd/p16kd/RasV12/ST
14% gel
