# Supplementary material for: Opposing activities of the Ras and Hippo pathways converge on regulation of YAP protein turnover
Source: EMBO J. 2014 Sep 1;33(21):2447–57. doi: 10.15252/embj.201489385 (PMC4283404; doi:10.15252/embj.201489385)
Supplement: Supplementary file 7 [file embj0033-2447-sd7.pdf]

Supplementary Information

Figure S1: Different forms of Ras

K-, N- and H-Ras

K-Ras is commonly mutated in cancers, including colorectal cancers (TCGA Network 2012, *Nature* **487:** 330-337). K-Ras and N-Ras contain rare codons that reduce their translation efficiency copmpared to H-Ras (Lampson et al 2013 *Curr Biol* **23:** 70-75). Native H-Ras is more effective in cell-based assays and has traditionally been used in primary cell transformation assays (Hahn et al., 1999 *Nature* **400:** 464-468). Using versions of H-, N- and K-Ras in which some of the the rare codons were replaced (Lampson et al., 2013), we find that all three isoforms transformed primary human BJ cells.

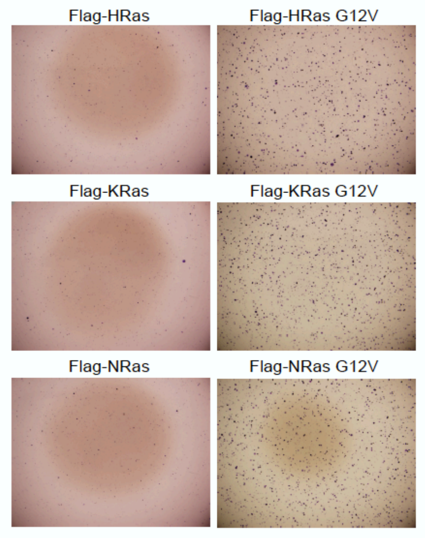

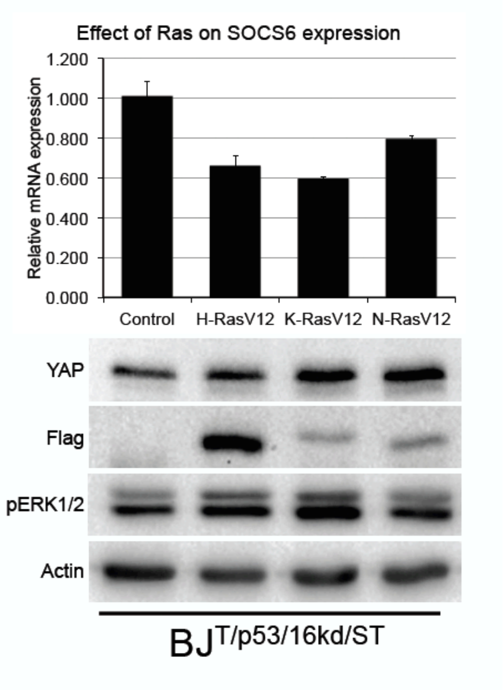

Comparison of the effects of H-, N- and K-Ras on SOCS6 mRNA expression. BJ cells were transduced to express G12V mutant forms of H-, N- and K-Ras, as above. SOCS6 mRNA level was reduced by all three forms of Ras. YAP protein level was increased. Note that K- and N-Ras were expressed at lower level than H-Ras, despite the codon optimization. The H-Ras lane contains less total protein (actin loading control).

Figure S2: effects of SOCS5/6 depletion

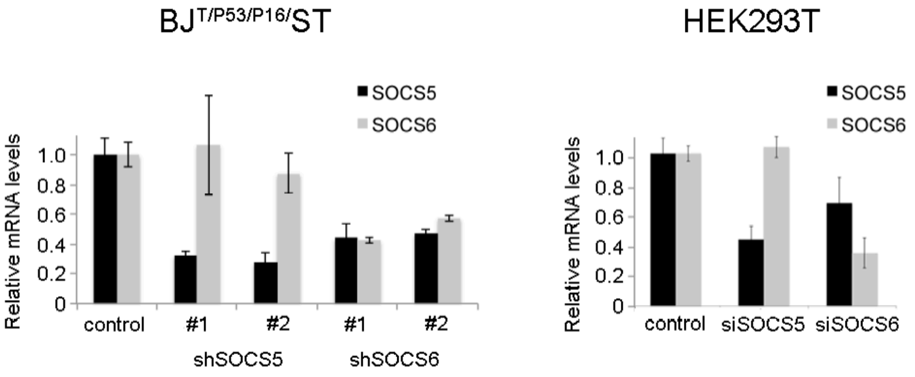

Left panel, two independent shRNAs targeting SOCS5 or SOCS6 were retrovirally transduced into $BJ^{T/p53/p16KD}$/ST cells expressing p53 and p16-shRNAs, small T antigen and hTert. Empty vector was used as a control. shRNA knockdown efficacy was quantified by real time quantitative RT-PCR. Data were normalized to GAPDH. We observed that SOCS6 depletion reduced both SOCS6 and SOCS5 mRNA levels, but SOCS5 depletion had little or no effect on SOCS6 levels. SOCS6 depletion also reduced the level of the unspliced SOCS5 primary transcript (not shown). The effect of SOCS6 depletion is therefore most likely an indirect effect on SOCS5 transcription, rather than a direct effect on SOCS5 mRNA. Data show the average of three independent experiments (error bars: ± SD).

Figure S3: images of xenograft tumors presented in Table 1

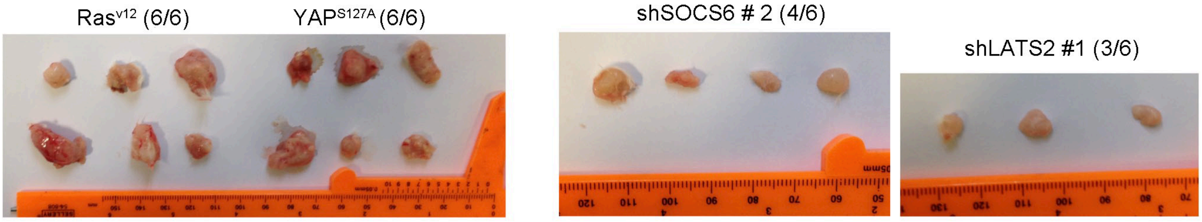

Primary $BJ^{T/p53KD/p16KD/ST}$ cells were transduced to express Ras^{V12} , YAP^{S127A}, shRNAS to deplete SOCS6, LATS2 or with empty vector as a control. Two million cells were injected subcutaneously adjacent to the legs of 4-5 week NOD-*scid Il2rg-/-* mice (Chen Q et al., 2009 *Proc Natl Acad Sci USA* **106:** 21783-21788). Left: Ras^{V12} and YAP^{S127A} tumors harvested after 6.5 weeks. Right panels: shSOCS6 and shLATS2 tumors harvested after 9 weeks. Note the difference in magnification.

Figure S4: Control for figure 2(B)

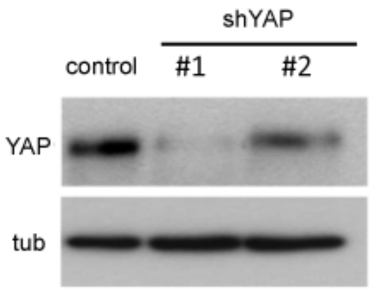

Immunoblot showing depletion of YAP protein following the shRNA treatment of BJ cells. Samples from Figure 2(B).

Figure S5: activation of endogenous YAP bypasses Ras^{V12}

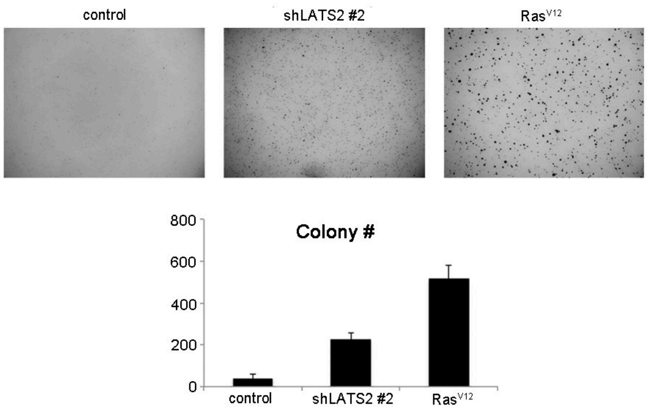

LATS2 depletion using a second independent shRNA supported colony formation in $BJ^{T/p53KD/p16KD/ST}$ cells without expression of Ras^{V12}. Histogram: colony formation from 3 independent experiments (average ± SD). Expands on Figure 2C.

Figure S6: Transformation efficiency of YAP^{S127A} in HMEC cells

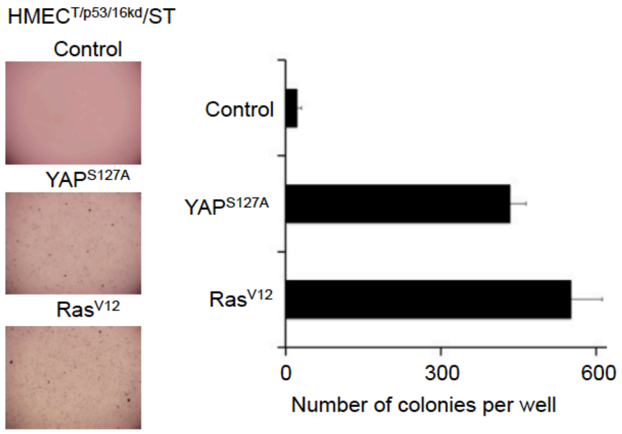

YAP1 was able to replace Ras^{V12} in transformation of primary human mammary epithelial cells ($HMEC^{T/p53KD/16KD/ST}$). $HMEC^{T/p53/p16KD}/ST$ cells were retrovirally transduced to express YAP^{S127A} or with empty vector as a control. Histogram: average colony numbers from 3 independent experiments (± SD).

Figure S7: SOCS6 acts on TAZ

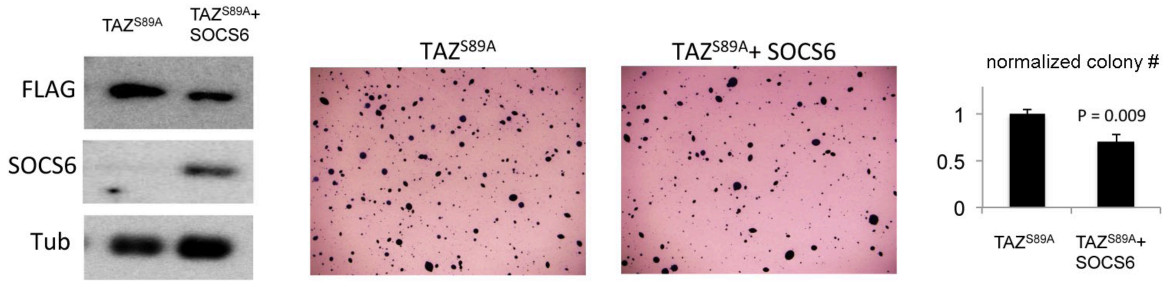

The LATS kinase resistant mutant form of TAZ, TAZ^{S89A}, is regulated by SOCS6 overexpression in $BJ^{T/p53/p16KD}$/ST cells. Left panel: Immunoblot analysis showing reduced FLAG-TAZ^{S89A} when SOCS6 was overexpressed. Tubulin was used as a loading control. Middel panels: representative microscopic images of soft agar assay in TAZ^{S89A}-expressing $BJ^{T/p53/p16KD}$/ST cells transduced with empty vector or HA-SOCS6. Right panels: Histogram of colony formation from three independent experiments (average ± SD). SOCS6 expression reduced colony formation drven by LATS-resistant TAZ ($p<0.01$, t-test).

Table S8: socs6 mRNA/DNA in colorectal cancer datasets

Data set	SOCS6
TCGA (DNA)	-1.3 fold, $p = 1 \times 10^{-51}$
Kurashina et al 2008 (DNA)	-1.2 fold, $p = 1 \times 10^{-11}$
Gaedcke et al 2010	-1.9 fold, $p = 2 \times 10^{-25}$
Hong et al 2010	-2.5 fold, $p = 9 \times 10^{-15}$
TCGA (mRNA)	-2.2 fold, $p = 1 \times 10^{-14}$
Skrzypczak et al 2010	-1.9 fold, $p = 2 \times 10^{-7}$
Ki et al 2007	-1.3 fold, $p = 4 \times 10^{-5}$
Kaiser et al 2007	-1.9 fold, $p = 3 \times 10^{-4}$

The status of SOCS6 DNA or mRNA level in a subset of colorectal cancer datasets that are available in Oncomine (www.oncomine.org). p-value and fold change are shown for cancer vs normal tissue. References available at www.oncomine.org.

Table S9: Survey of correlation between SOCS6 and AREG mRNA levels in cancer.

Cancer types	correlation coefficient (AREG VS SOCS6)	p values	reference
Uterine Corpus Endometrioid Carcinoma_TCGA_RNAseq	0.1	0.06	1
CSCC and EA	0.07	0.45	1
Lung Squamous Cell Carcinoma_TCGA_RNAseq	0.06	0.32	2
Breast Invasive Carcinoma_TCGA_RNAseq	0.04	0.2	3
Acute Myeloid Leukemia	-0.01	0.87	4
Thyroid Carcinoma	-0.04	0.51	1
Prostate Adenocarcinoma	-0.06	0.41	1
Lung Adenocarcinoma_RNAseq	-0.06	0.35	1
Kidney Renal Clear Cell Carcinoma_TCGA2_RNAseq	-0.06	0.17	5
Brain Lower Grade Glioma_RNAseq	-0.08	0.25	1
Ovarian Serous Cystadenocarcinoma_TCGA_microarray	-0.1	0.03	6
Bladder Cancer	-0.14	0.31	7
Glioblastoma Multiforme_microarray	**-0.22**	**7.03E-07**	1
Skin Cutaneous Melanoma	**-0.24**	**6.95E-05**	1
Colon and Rectum Adenocarcinoma_TCGA_RNAseq	**-0.27**	**1.62E-05**	1

mRNA expression data were extracted from cBioPortal (1). For cancer types with multiple datasets, we selected the set with largest sample size for analysis. cBioPortal z scores are determined by comparing mRNA expression to the distribution in a reference population. The reference population is either mRNA expression in normal adjacent tissues or expression data from all tumors diploid for the gene. Partial correlational analysis was employed to calculate the association between SOCS mRNA and AREG mRNA levels in each cancer type, with the effect of YAP mRNA level removed (Baba et al, 2004 *Australian & New Zealand Journal of Statistics* **46**, 657-664). False discovery rate (q value) was used to correct for multiple comparisons. $q \leq 0.05$ was used as the cutoff for statistical significance. Significant inverse correlation was observed between SOCS6 and AREG mRNAs levels in colon and rectal adenocarcinoma ($r=-0.27$, $p<2E^{-5}$, false discovery rate $q=1E^{-4}$) and skin cutaneous melanoma ($r=-0.24$, $p<7E^{-5}$, $q=3E^{-4}$), and in glioblastoma multiforme ($r=-0.22$, $p<7E^{-7}$, $q=1E^{-5}$). Differences in YAP mRNA levels were corrected for in this analysis, hence the inverse correlation between SOCS6 and YAP targets is likely due to change in YAP protein activity. Note that this correction removes the effects of increased YAP mRNA in some cancers, and so likely underestimates the true magnitude of the increase in AREG vs SOCS6.

1) Gao J, Aksoy BA, Dogrusoz U, Dresdner G, Gross B, Sumer SO, Sun Y, Jacobsen A, Sinha R, Larsson E, Cerami E, Sander C, Schultz N (2013) Integrative analysis of complex cancer genomics and clinical profiles using the cBioPortal. *Sci Signal* **6:** pl1
2) TCGA Network (2012a) Comprehensive genomic characterization of squamous cell lung cancers. *Nature* **489:** 519-525
3) TCGA Network (2012b) Comprehensive molecular portraits of human breast tumours. *Nature* **490:** 61-70
4) TCGA Network (2013b) Genomic and epigenomic landscapes of adult de novo acute myeloid leukemia. *New Eng J Med* **368:** 2059-2074
5) TCGA Network (2013a) Comprehensive molecular characterization of clear cell renal cell carcinoma. *Nature* **499:** 43-49
6) TCGA Network (2012) Comprehensive genomic characterization of squamous cell lung cancers. *Nature* **489:** 519-525
7) Iyer G, Al-Ahmadie H, Schultz N, Hanrahan AJ, Ostrovnaya I, Balar AV, Kim PH, Lin O, Weinhold N, Sander C, Zabor EC, Janakiraman M, Garcia-Grossman IR, Heguy A, Viale A, Bochner BH, Reuter VE, Bajorin DF, Milowsky MI, Taylor BS, Solit DB (2013) Prevalence and co-occurrence of actionable genomic alterations in high-grade bladder cancer. *J Clin Oncol* **31:** 3133-3140
8) CGAR Network (2008) Comprehensive genomic characterization defines human glioblastoma genes and core pathways. *Nature* **455:** 1061-1068

Figure S10: RNAic   mediated depletion of SOCS6 in cancer cell lines

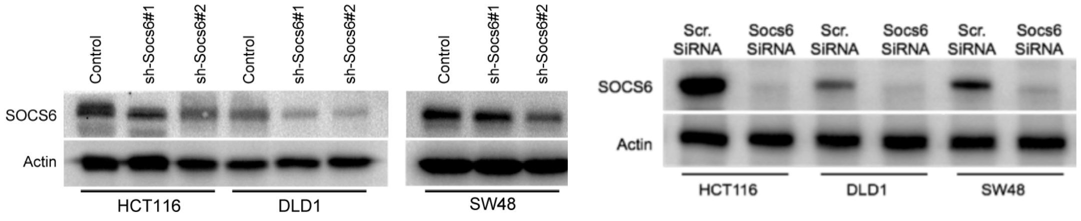

Immunoblots showing shRNA-mediated (left) and siRNA-mediated (right) depletion of SOCS6 in the indicated cancer cell lines.

Figure S11: Depletion of EGFR limits YAP mediated colony formation

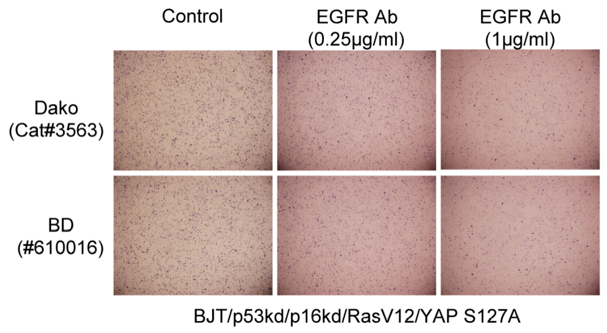

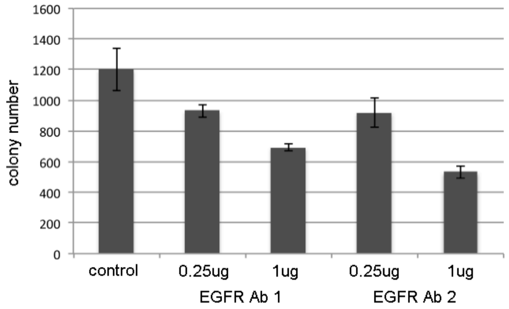

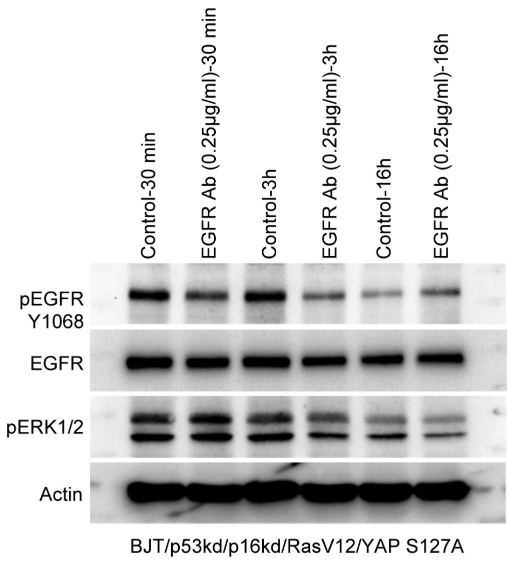

Primary BJ$^{T/p53KD/p16KD/ST}$ cells were transduced to express YAP^{S127A} and treated with anti-EGFR. Anti-EGFR treatment caused a dose-dependent decrease in colony formation. The histogram shows average ± SD (ANOVA $p<0.05$ comparing untreated vs 0.25μg/ml; $p<0.01$ comparing untreated vs 1μg/ml). Anitbody-containing medium was refreshed twice at 4 day intervals. Ab-treatement caused a transient decrease in EGFR activation, visualized with anti-pY1068 (left panel). Ab1 was from DAKO (cat# 3563). Ab2: the first treatment was with anti-EGFR from BD (cat# 10016); treatments 2 and 3 were with the DAKO antibody.
